# Bacterial Succession on Sinking Particles in the Ocean's Interior

**DOI:** 10.3389/fmicb.2017.02269

**Published:** 2017-11-24

**Authors:** Erik A. Pelve, Kristina M. Fontanez, Edward F. DeLong

**Affiliations:** ^1^Department of Cell and Molecular Biology–Molecular Evolution, Biomedical Center, Uppsala University, Uppsala, Sweden; ^2^Department of Civil and Environmental Engineering, Massachusetts Institute of Technology, Cambridge, MA, United States; ^3^Daniel K. Inoue Center for Microbial Oceanograpy: Research and Education, Department of Oceanography, University of Hawaii at Manoa, Honolulu, HI, United States

**Keywords:** marine bacteria, oligotrophic gyre, metagenomics, marine particles, sediment trap, biological pump, single cell genomics

## Abstract

Sinking particles formed in the photic zone and moving vertically through the water column are a main mechanism for nutrient transport to the deep ocean, and a key component of the biological carbon pump. The particles appear to be processed by a microbial community substantially different from the surrounding waters. Single cell genomics and metagenomics were employed to describe the succession of dominant bacterial groups during particle processing. Sinking particles were extracted from sediment traps at Station Aloha in the North Pacific Subtropical Gyre (NPSG) during two different trap deployments conducted in July and August 2012. The microbial communities in poisoned vs. live sediment traps differed significantly from one another, consistent with prior observations by Fontanez et al. (2015). Partial genomes from these communities were sequenced from cells belonging to the genus *Arcobacter* (commensalists potentially associated with protists such as Radiolaria), and *Vibrio campbellii* (a group previously reported to be associated with crustacea). These bacteria were found in the particle-associated communities at specific depths in both trap deployments, presumably due to their specific host-associations. Partial genomes were also sequenced from cells belonging to *Idiomarina* and *Kangiella* that were enriched in live traps over a broad depth range, that represented a motile copiotroph and a putatively non-motile algicidal saprophyte, respectively. Planktonic bacterial cells most likely caught in the wake of the particles belonging to *Actinomarina* and the SAR11 clade were also sequenced. Our results suggest that similar groups of eukaryote-associated bacteria are consistently found on sinking particles at different times, and that particle remineralization involves specific, reproducible bacterial succession events in oligotrophic ocean waters.

## Introduction

Sinking particles produced in the photic zone that transport nutrients to the deep ocean are an important sink for carbon dioxide from the atmosphere. The particles in the upper layers of the ocean are formed from a complex interplay between abiotic factors, eukaryotic cells, and phototrophic and heterotrophic bacteria. As the particles sink they are processed by a distinct community of microbes, and the nutrients released are made available to other trophic levels of the ecosystem through the microbial carbon pump (Jiao et al., 2010). Sinking particles in the ocean can be viewed as discrete loci that share some properties to island habitats, as isolated, discrete environments with different environmental conditions than are found in the surrounding water. As such, they are hot spots for microbial activity and are important hubs in the food web, with anaerobic and aerobic metabolic micro-niches (Honjo et al., 2008).

Particles exist in the ocean in a range of forms, sizes, and compositions (Simon et al., 2002). Many remain in the photic zone until they are disaggregated, but some particles sink, mainly macroaggregates composed of algae, diatoms, dinoflagellates, fecal material, and minerals derived from eukaryotic shells and similar remains. Microbial communities on particles have sometimes been shown to be significantly different from that of the surrounding water (DeLong et al., 1993; Allen et al., 2013), but they are diverse and heterogeneous and are still not fully understood. For example, the identity and relative abundance of eukaryotes that produce particulate materials is still largely unknown, as is the specificity of their bacterial associations and depth distributions, and how species interactions may affect particle degradation. How initial microbial communities found on sinking particles might change during transformation of particulate organic material (POM) to dissolved organic material (DOM) also remains to be fully determined.

Methods typically used to separate different size fractions from one other can present challenges in identifying authentic particle-associated communities (Grossart, 2010). Additionally, microbial composition and degradation processes on sinking particles may differ in their degradation dynamics as compared to marine debris that are stationary in the water column (Honjo et al., 2008). Yet another consideration are the methods used to characterize microbial components on the sinking particles. PCR-based approaches that use functional or taxonomic indicator genes can provide an extensive fingerprint of the community composition, but are also vulnerable to well known biases. Shotgun metagenome sequencing has the potential to provide a more even description of the community, but requires larger sequencing effort, and may miss rare members of the community. Single cell genomics can provide partial genomes and more precise identification of gene origins, albeit with lower overall throughput. In this study, we sought to combine several approaches (single cell genomics and metagenomics) to overcome the individual shortcomings of either of these techniques used alone.

A recent study demonstrated that the initial microbial community on sinking particles in the oligotrophic North Pacific was dominated by bacterial groups associated with eukaryotes, while copiotrophic bacterial species dominated in sediment traps in which particle degradation occurred (Fontanez et al., 2015). Metagenomes from particles caught in sediment traps contained DNA from microbial communities distinct from the surrounding seawater. Poisoned traps containing a fixative that preserves the microbes and their nucleic acids as they enter the trap, were rich in sequences associated with eukaryotes, viruses, and bacteria (including the functions pathogenicity, anaerobic metabolism, and chitin degradation). Live traps, that allow the particle degrading community to enrich *in situ*, were rich in bacterial taxonomic and functional genes known to be involved in degradation of the DOM released during POM processing, as well as bacteria associated with eukaryotes.

In this study, we combined the metagenomes obtained from a previous report (Fontanez et al., 2015) with corresponding metagenomes collected on a different set of deployments and single cell genomes that represented six dominant bacterial genera found in the traps, to improve our understanding of the major microbial players associated with the initial and processed sinking particles in the oligotrophic North Pacific Subtropical Gyre (NPSG). Single cell genomes combine taxonomic marker genes with functional genes, allowing for specific predictions of ecological function. Based on earlier studies, we postulated that dominant bacterial genera found in the initial sinking community on particles (poisoned traps), would encode genes indicative of their association with eukaryotes. Similarly, we postulated that bacterial genera enriched in the “processed” community (live traps), would be enriched in genes coding for particle degradation, motility, chemotaxis, and DOM degradation. To test these hypotheses, we combined results from several different trap deployments, using both metagenomics, and single cell sequencing, to more completely describe the characteristics of microbes on sinking materials and in processed POM, and how they varied at different times and as a function of depth. Using the same data, we also tested the hypothesis that particle-associated bacteria are typically recruited onto sinking particles mainly in the photic zone, and are subsequently present on the particles throughout the water column at greater depths.

## Materials and methods

Metagenomes collected from poisoned and live traps from the July HD5 cruise at station ALOHA in the NPSG (22°48.5 N, 158°3.02 W) in 2012 were analyzed by Fontanez et al. (2015). In the current study, these metagenome data were combined with a different set of metagenomes collected in August of the same year in a separate set of trap deployments on cruise HD9, in addition to determination of single cell genomes collected from the sediment traps (this study).

### Sampling

**Figure 1:**
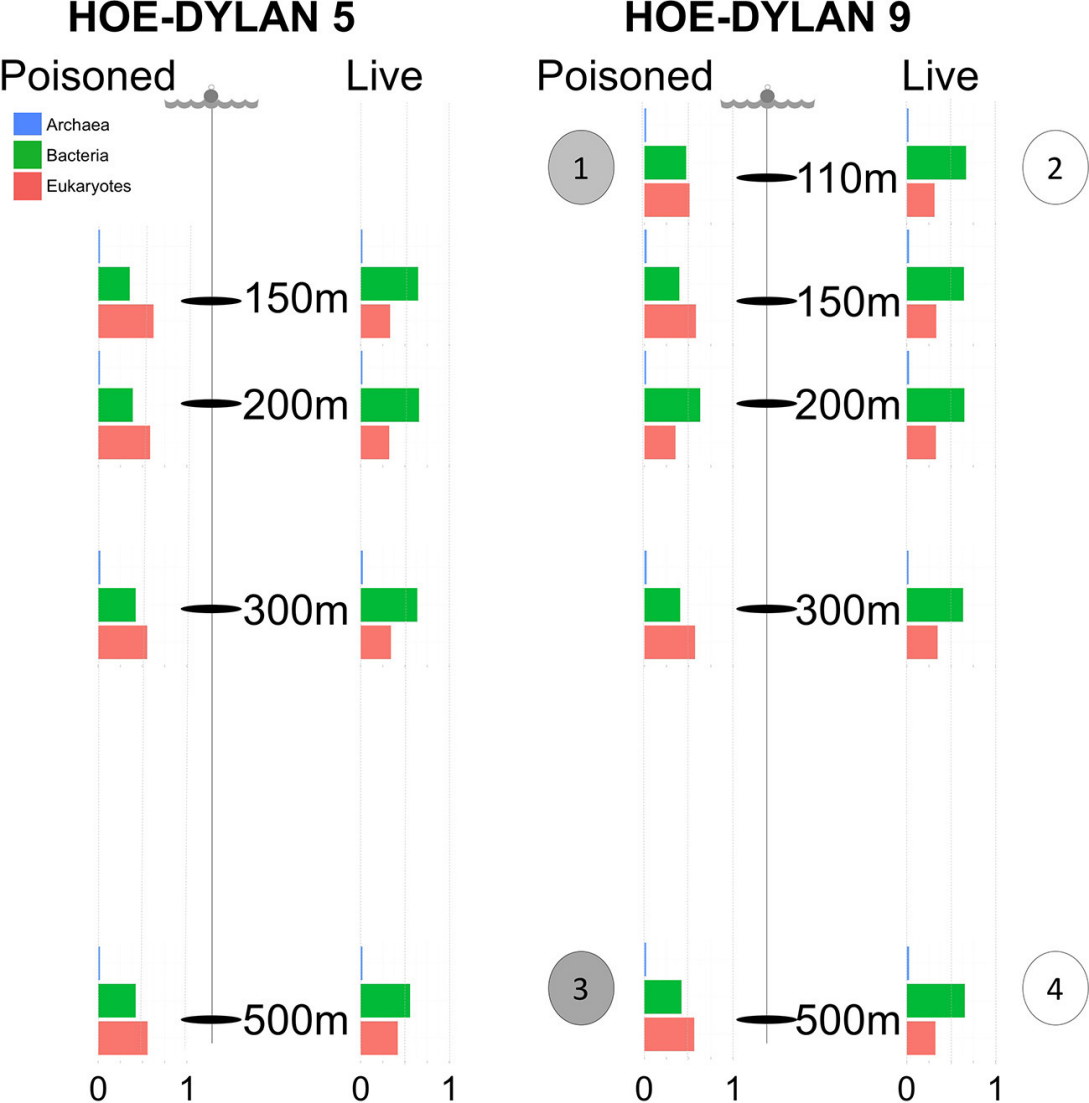
Samples for metagenomic sequencing were collected from poisoned and live sediment traps placed at five different depths during two cruises in July and August 2012, representing the initial and enriched particle processing community, respectively. The bar charts display relative proportion of SSU sequences identified in the metagenome and annotated as Archaea, Bacteria, and Eukaryotes at each depth in each trap. Single cell genomes were collected from the following traps: (1) Poisoned, 110m: 2 × Vibrio. (2) Live, 110m: 2 × Idiomarina, 2 × Kangiella. (3) Poisoned, 500m: 2 × Arcobacter, 1 × SAR11 clade. (4) 1 × Actinomarina.

Sinking particles were collected during the 2012 HD5 and HD9 cruises. Two sets of free drifting sediment traps were placed at depths of 110, 150, 200, 300, and 500 m, both sets containing ~1.8 L of sterile filtered seawater (0.2 μm), adjusted to a density of 1.05 g/cc with NaCl (Figure 1). The poisoned traps contained the fixative RNAlater (Ambion, Carlsbad, CA, USA), designed to prevent biological degradation of material that enters the traps. A 335 μm nylon screen (Nitex) was placed on top of the tubes to prevent larger zooplankton from entering the trap. The traps from the HD5 cruise were deployed in July 14 and recovered in July 26 2012 and the traps from the HD9 cruise were deployed in August 27 and recovered in September 12 2012 (see Fontanez et al., 2015 for methodological detail).

### Sample processing

About 500 mL of seawater from the top of each tube was removed to limit contaminating traces of the surrounding sea water. In the HD5 samples, particles in the 0.2–335 μm fraction were collected on a Sterivex filters (EMD Millipore, Billerica, MA, USA). In the HD9 samples, particles were collected by serial filtration in two different size fractions, first in the 5.0–335 μm size fraction, and next in the 0.2–5.0 μm size fraction. After filtration, filters were stored in 1.5 mL RNAlater and frozen at −80°C (see Fontanez et al., 2015 for details). Samples for single cell analyses were collected from the bottom of live and poisoned sediment traps at 110 and 500 m. A total of 1.6 mL of the unfiltered sediment trap solution was added to 400 μL of 50% glycerol in 1X phosphate buffered saline, flash frozen, and stored at −80°C until single cell genome amplification at the Bigelow Single Cell Genomics Center.

### Metagenomic sequencing and processing

DNA from the traps were purified and sequenced (as described in detail in the Supplemental information of Fontanez et al., 2015). In short, DNA was purified from filters with the Mobio Powerwater DNA Isolation kit (Mobio, Carlsbad, CA). Libraries were prepared using the Nextera XT DNA sample preparation protocol (Illumina, San Diego, CA, USA) and sequenced on an Illumina MiSeq instrument, with 10 dual indexed samples pooled for each sequencing run. Library quality and fragment size range were evaluated with a Bioanalyzer 2100 (Agilent Technologies, Santa Clara, CA, USA). All metagenomic data are available at NCBI under bioproject number PRJNA270248.

### Single cell genomic sequencing

Single cells were isolated through FACS flow cytometry at the Bigelow Single Cell Genomics Center (as described in Woyke et al., 2009). DNA was amplified with by multiple displacement amplification (MDA) and single amplified genomes (SAGs) were screened with PCR using primers targeting bacterial and archaeal 16S genes (27F:AGRGTTYGATYMTGGCTCAG/907R:CCGTCAATTCMTTTRAGTTT; Arc_344F:ACGGGGYGCAGCAGGCGCGA/Arc_915R:GTGCTCCCCCGCCAATTCCT). PCR products were Sanger sequenced using the BigDye 3.1 kit at the ABI 3730 DNA analyzer, and the sequences quality controlled with the software Sequencher (Sequencher® version 4.10.1 sequence analysis software, Gene Codes Corporation, Ann Arbor, MI, USA http://www.genecodes.com) and aligned to the SILVA 115 database using LAST (Zhang et al., 2000) with a cutoff of lastscore 50, *e*-value 0.01.

Ten SAGs were selected for whole genome sequencing. The selected SAGs were chosen to represent different trap conditions and time points in particle progression. From the poisoned traps, two SAGs each were chosen belonging to the genera Arcobacter and Vibrio. Both were found to be significantly enriched in poisoned traps compared to the surrounding sea water, and both harbored genes that were highly enriched in the poisoned traps (Fontanez et al., 2015). Both genera are also known to include strains closely associated with eukaryotic cells of the kind that are suggested to be important for the early stages of particle processing. From the live traps, two SAGs each belonging to the genera Idiomarina and Kangiella were chosen. Both genera are enriched in live traps compared to poisoned traps and the surrounding sea water (Fontanez et al., 2015). Idiomarina is commonly enriched in mesocosms and Kangiella is related to the hydrocarbon degrader Oceanospirillales, which suggests an important function in the latter stages of particle processing. In addition, two SAGs were selected that did not represent dominant strains in the particle communities—one belonging to the SAR11 clade and one to the newly described genus Actinomarina. These SAGs were selected to shed light on their presence in the particles and to expand on the known genomic diversity of these groups.

For all sets of duplicate SAGs from the same genus, the pair of SAGs with the highest SSU nucleotide identity was chosen to allow for comparisons of closely related genomes. The selected SAGs were sequenced using Illumina NextEra and MiSeq. The libraries were quality controlled using Bioanalyzer 2100 (Agilent Technologies, Santa Clara, CA, USA).

### Metagenomic analysis

Reads from the two different size fractions (0.2–0.5 and 0.5–335 μm) from the HD9 cruise were pooled together and treated in aggregate, for comparison to the 0.2–335 μm samples from the HD5 cruise. The reads were trimmed and processed as described by Fontanez et al. (2015) and queried against the SILVA 123, Refseq64, and KEGG (September 2013) databases using LAST (0.1.0; Score penalty: 500; Initial match multiplicity (–m): 10; Zhang et al., 2000). Selective enrichment of Operational Taxonomic Units (OTUs) in metagenomes were analyzed using the R packages phyloseq (McMurdie and Holmes, 2013), vegan (Oksanen et al., 2016) and DESeq2 (Love et al., 2014). In DESeq2, the regularized log transformation was used to normalize the samples (blind = FALSE), taking into account different sample sizes. The complete dataset (sequences belonging to all domains of life) was used for normalization, comparable to what was described in Fontanez et al. (2015). Abundance plots of 2^∧^(transformed value) was created in the phyloseq R package, where principal coordinate analysis was used to ordinate the normalized sequences. The Vegan R package was used to detect significantly divergent clusters (*p* < 0.05) with the Adonis function. The DESeq2 package was used to statistically infer divergent groups based on taxonomic inference (using the Wald function) from the Silva and Refseq databases, as well as functional genes based on the KEGG database. Traps were compared in an experimental design where poisoned and live traps were compared over both cruises with traps at all depths used as biological replicates. Deep (300, 500 m) and Shallow traps (110, 150, and 200 m) were compared with traps from both cruises and both treatments as biological replicates. Cruises were compared with traps of all depths and both treatments as biological replicates (Table S5). Significantly enriched OTUs were extracted at a *p* value of 0.01 (see Fontanez et al., 2015 for details.).

### Single cell data processing

Single cell datasets were analyzed on the Galaxy platform. The Illumina reads were filtered using trimmomatic (Seed mismatches: 2; Palindrome clip threshold: 40; Sliding window size: 4 Average quality required: 15; Minimum quality leading/trailing bases: 5 Minimum length read: 45; Lohse et al., 2012). Paired reads were assembled using SPADES 3.0 with BWA-mediated MismatchCorrector (K-mers: 21,33,55; Bankevich et al., 2012). Assembly quality was assessed using QUAST (Gurevich et al., 2013) and completeness predicted using a set of 139 marker genes (Rinke et al., 2013). Scaffolds were annotated using PROKKA (transl_table: 11, Similarity *e*-value cut-off: 1e-06; Cuccuru et al., 2014; Seemann, 2014) and LAST (0.1.0; Score penalty: 500; Initial match multiplicity (–m): 10; Zhang et al., 2000) against the databases Refseq64 and KEGG (September 2013), and the carbohydrate-active enzymes database (CAZymes, October 2017; Lombard et al., 2014).

Related genes to the SAG SSU RNA genes predicted from PROKKA were identified in the SILVA 123 database. The sequences were filtered to 1,000 bp, clustered to 95% similarity with usearch (Edgar, 2010), aligned using linsi (with the parameters set to auto) in the MAFFT package (Katoh and Standley, 2013), cropped with BMGE (DNAPAM1:2; Criscuolo and Gribaldo, 2010), and a phylogenetic tree was produced with IQ-tree (fast bootstrapping, 1,000 replicates, model selected by the TEST algorithm and reported for each tree in Figure S4; Nguyen et al., 2015). The trees were refined in the software FigTree (http://tree.bio.ed.ac.uk/software/figtree/).

Average nucleotide identity and tetranucleotide frequency were used to determine relationship between the SAGs and reference genomes using the script pyani (https://github.com/widdowquinn/pyani). Shared genes between related genomes were determined using reciprocal best blast hit (60% similarity, *e-*value 1e-10). Shared genes between multiple genomes were defined from shared homologous orthoMCL groups (Chen et al., 2006). Metagenomic reads were recruited to the SAGs with blastn (cutoff: 200 bp, 90% sequence similarity) and used to estimate SAG abundance between depths, treatments, and cruises. Relative read recruitment as aspects of treatment, depth, and cruise were compared to each other with an ANOVA test, with the other samples as biological replicates in the same experimental design as described for the metagenome. Reads annotated to the same genus as the SAGs in the metagenomes of the same traps as where the SAGs were isolated were selected and gene orthology (on KO level) annotated. These annotations were compared to the annotations from the SAGs as well as corresponding annotations from the closest fully sequenced genome (to compensate for missing regions from the SAGs) to determine to which extent the SAGs represent their genera in the traps. Reference genomes: *Arcobacter nitrofigilis* (NC_014166); *Vibrio campbellii BAA1116* (NC_009783; NC_009784; NC_009777); *Idiomarina loihiensis* (NC_021286); *Kangiella koreensis* (NC_013166); *Actinomarina minuta* (KC811150); *Alpha proteobacteria HIMB5* (NC_018643).

All single cell data are available at NCBI under bioproject number PRJNA270248 with the following nomenclature:

Uncultured Actinomarina HD9-500 m-PIT-SAG01—Actinomarina
Uncultured Arcobacter HD9-500 m-PIT-SAG02—Arcobacter1
Uncultured Arcobacter HD9-500 m-PIT-SAG03—Arcobacter2
Uncultured Idiomarina HD9-110 m-PIT-SAG04—Idiomarina1
Uncultured Idiomarina HD9-110 m-PIT-SAG05—Idiomarina2
Uncultured Kangiella HD9-110 m-PIT-SAG06—Kangiella1
Uncultured Kangiella HD9-110 m-PIT-SAG07—Kangiella2
Uncultured SAR11clade HD9-500 m-PIT-SAG08—SAR11_clade
Uncultured Vibrio HD9-110 m-PIT-SAG09—Vibrio1
Uncultured Vibrio HD9-110 m-PIT-SAG10—Vibrio2

## Results

Single cell genomes from the HD9 cruise and metagenomes from the HD5 and HD9 cruises were sequenced and analyzed to enable comparisons of the microbial assemblages on sinking particles caught in poisoned and live sediment traps deployed in the NPSG (22°48.5 N 158°3.02 W).

### Samples

Metagenome samples were collected from traps placed at 150, 200, 300, and 500 m depth during two cruises (HD5 and HD9) in July and August 2012, as well as at 110 m for the second cruise (Figure 1, Table 1, Table S5). Two types of traps were deployed: (1) poisoned traps which contained filtered seawater adjusted to a density of 1.05 g/cc with brine solution, combined with a fixative to preserve nucleic acids of the initial community of the sequestered particles; and (2) live traps, containing only the brine solution that allowed living microbial communities to further process particles collected in the traps (see section Materials and Methods and Fontanez et al., 2015). Single cells were isolated and their genomes were sequenced from both live and poisoned traps from 110 to 500 m depth during the HD9 cruise.

**Table 1:** Significantly enriched operational taxonomic units (OTUs) in the metagenomes (*p* value < 0.01).

**Database**	**Domain**	**Poiss_vs_Live**	**Live_vs_Poiss**	**Deep_vs_Shallow**	**Shallow_vs_Deep**	**HD5_vs_HD9**	**HD9_vs_HD5**
Refseq	Archaea	1	3	0	0	0	0
	Bacteria	31 (4)	213 (47)	8 (2)	4	0	5 (2)
	Eukaryotes	313 (205)	2	0	0	17	1
	Virus	32 (10)	1	0	0	0	0
Silva	Archaea	0	2 (2)	0	0	0	0
	Bacteria	3 (3)	28 (15)	1	0	0	2 (2)
	Eukaryotes	2 (1)	40 (36)	1	0	0	0
Kegg		3054 (2940)	347 (200)	19 (14)	2 (1)	0	2 (2)

*Numbers in parenthesis indicate enriched OTUs with log2-fold change > 2*.

### Metagenomes

**Figure 2:**
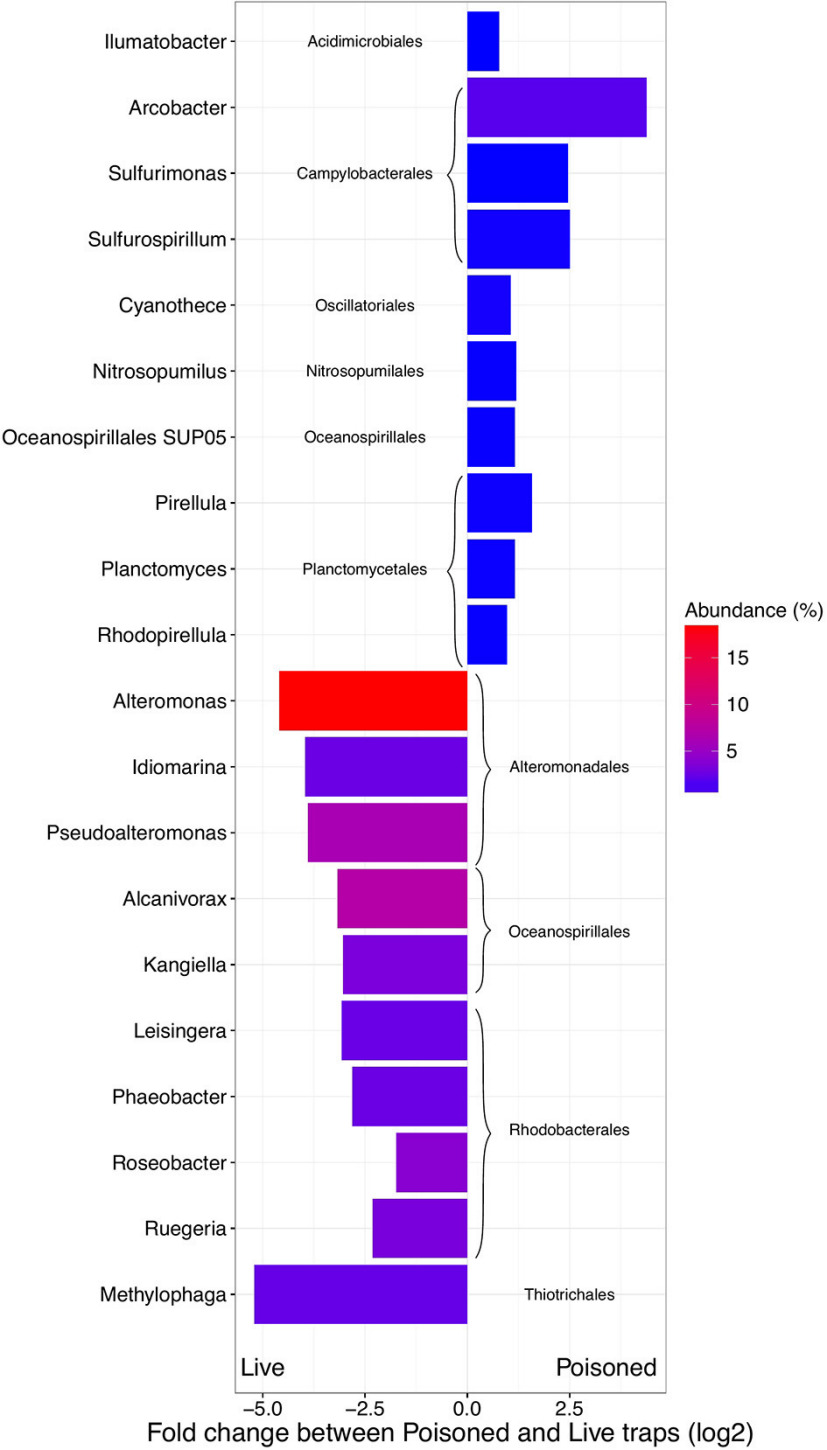
Difference in abundance (expressed as fold change between normalized taxonomic counts of read matches to the RefSeq database) in the initial and processed community for the 10 most abundant significantly enriched archaeal and bacterial genera in poisoned and live traps, respectively. The color represent average abundance in all traps (expressed as percentage of mean normalized counts in all traps).

Previously reported observations for samples collected during the HD5 cruise (Fontanez et al., 2015) were compared to processed and analyzed samples from the HD9 cruise 1 month later. Specifically, the metagenomes in the poisoned and live traps displayed distinct differences in microbial assemblage compositions. This was evident in KEGG, Refseq, and Silva database annotations (Figures 1, 2, Table 2, Table S1, Figure S1). There were only slight differences between traps of the same treatment from different depths (i.e., shallow poisoned traps compared to deep poisoned traps and shallow live traps compared to deep live traps).

**Table 2:** Assembly statistics of single cell genomes.

	**Initial community**				**Processed community**				**Planktonic**	
	**Arcobacter1**	**Arcobacter2**	**Vibrio1**	**Vibrio2**	**Idiomarina1**	**Idiomarina2**	**Kangiella1**	**Kangiella2**	**Actinomarina**	**SAR11-clade**
HD9 trap	500P	500P	110P	110P	110L	110L	110L	110L	500L	500P
Reads	693926	820791	898088	925567	821621	908546	828156	775789	729489	804475
Scaffolds	18	56	68	42	8	11	6	31	9	37
Total length	162008	397249	917768	1265-273	325153	597848	238196	1421-680	257233	361876
Largest scaffold	24156	56862	60299	167359	106931	163684	188967	240315	59530	28337
GC (%)	29.72	30.57	43.49	44.95	46.37	47.35	44.38	43.45	33.09	30.06
N50	15811	19010	26108	63133	72189	143629	188967	75140	41063	17212
L50	4	7	12	7	2	2	1	5	3	9
Estimated recovery (%)	3	20	6	33	8	20	13	61	22	30
Coding density (%)	78	86	79	84	94	94	90	90	96	91
Median intergenic length (bp)	89	36	114	94	19	16	63	60	2	3
rRNA	3	2	3	3	3	3	3	3	3	2
tRNA		14	14	29	4	8	10	21	13	11
tmRNA				1				1		
CDS	148	408	867	1211	334	581	244	1343	288	373
Hypothetical	44	41	68	62	80	29	239	32	251	237
CAZy genes		13	25	50	7	15	5	23	9	12
Novel genes		52	38			1		30	52	3

The possibility exists that differences between the HD5 an HD9 filtration methods (HD5, whole sample filtration, extraction, and sequencing, vs. HD9 serial filtration, extraction, and sequencing of two different size fractions separately, followed by *in silico* pooling of the two fractions) could introduce methodological differences in abundance estimates between the two different cruises. However, since very few differences were detected between the HD5 and HD9 results when the same sample treatments (poisoned or dead at the same depths) were compared, the different methods used did not appear to have compromised the overall results, at the level of resolution studied (see below).

Only a small number of OTUs were significantly different between the cruises (Table S1). Eukaryotes and viruses were more commonly observed and had higher relative abundance in poisoned traps compared to live traps (Figure 1). Bacteria were common in both sets of traps and relatively few sequences in any trap belonged to archaea. At a functional level, there were groups of genes involved in energy metabolism that appeared significantly enriched in deep, poisoned traps, including genes for carbon metabolism (K00177, K01846), a sulfite reductase (K00392), and genes involved in cofactor biosynthesis (K02188, K02190, K03404, K02293) (Figure S2). This might suggest specific life styles specifically enriched for growth among deep-dwelling eukaryotes and their associated bacteria.

### Single cell genomes

**Figure 3:**
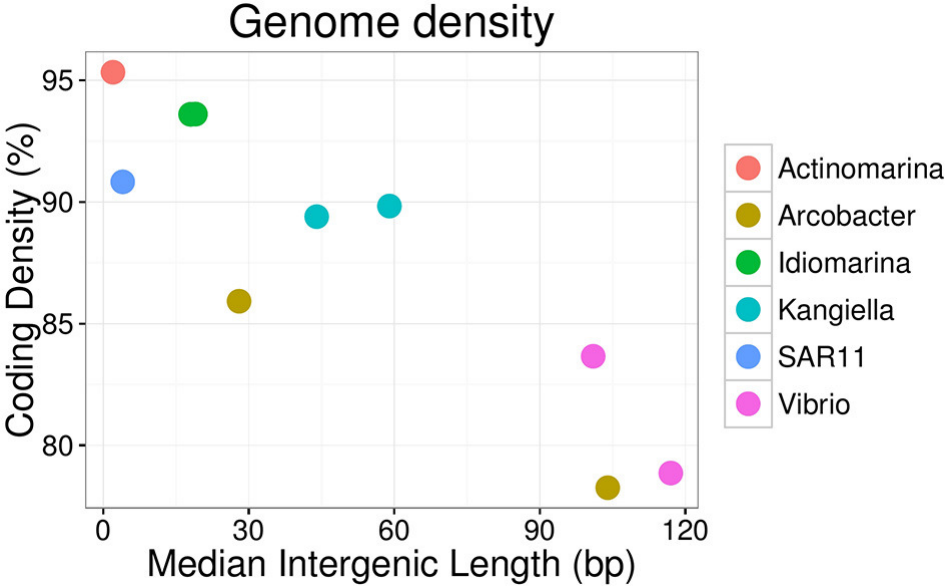
Genome density of SAGs, expressed as coding density (percentage of coding genome sequence out of total SAG sequence) and median intergenic length of scaffolds of the SAG.

**Figure 4:**
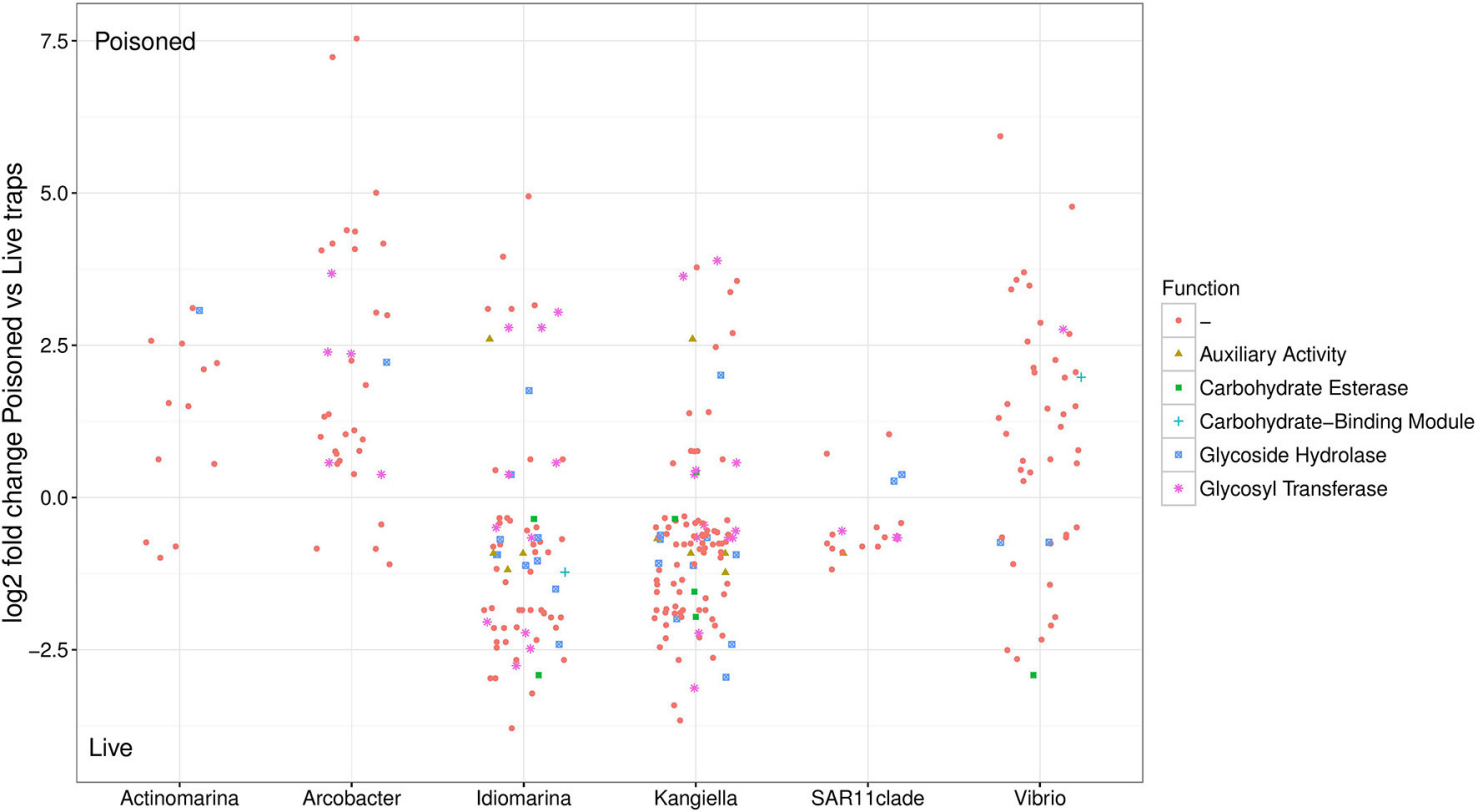
Relative abundance of SAG encoded genes in poisoned compared to live traps. Log2-fold change cutoff: 0.1. Functional categories from the Carbohydrate-Active enZYmes (CAZy) Database.

**Figure 5:**
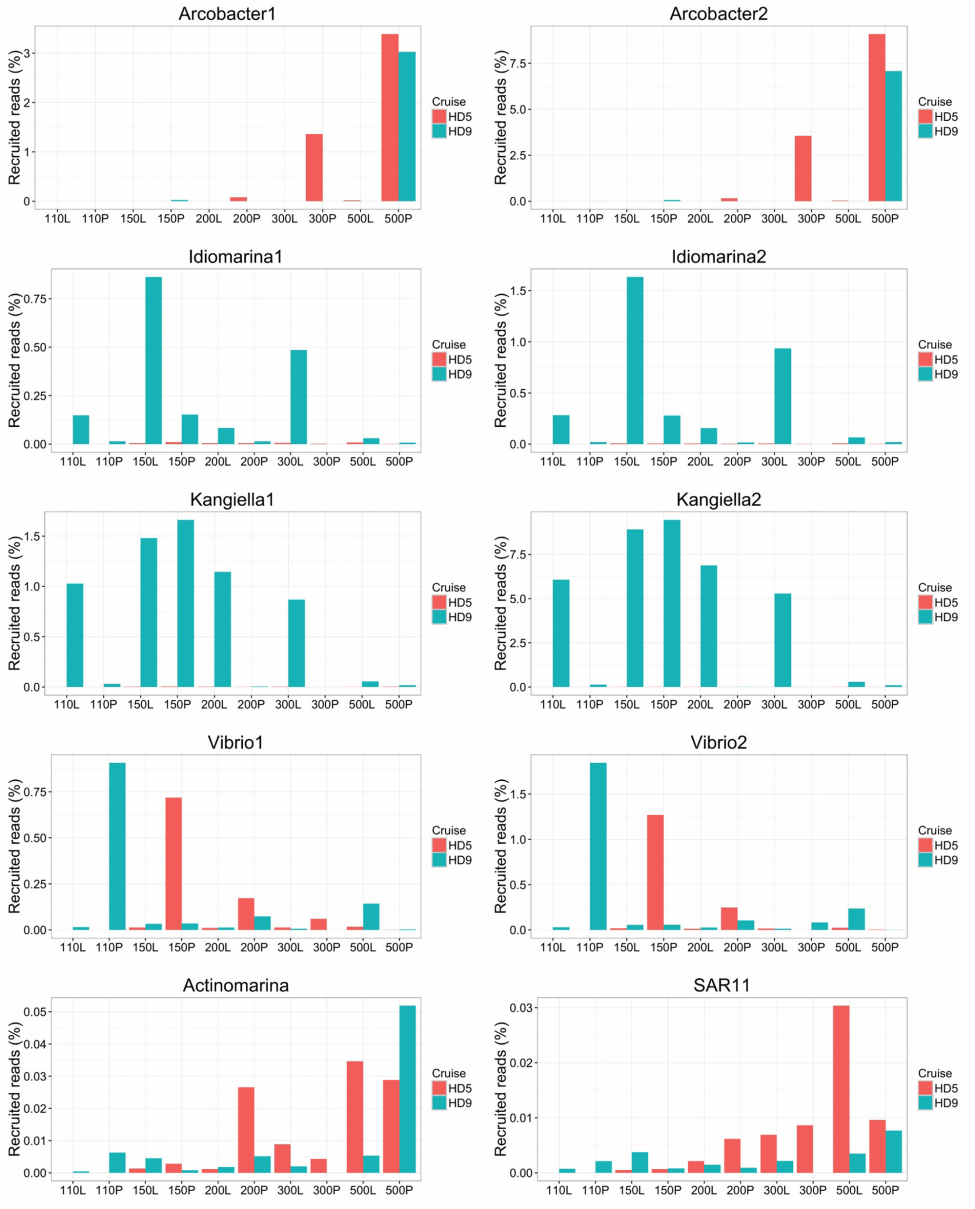
Relative abundance of SAGs in the HD5 and HD9 traps. Percentage of blastn-recruited metagenomic bacterial reads with 99% identity and 200 bp cutoff. Percentage of recruited reads from poisoned and live traps were compared with ANOVA using traps of different depth and from different cruises as biological control. Deep and Shallow traps and traps from the two cruises, respectively, were compared in the same way. The Arcobacter SAGs were significantly divergent between traps of different treatment and depth and the kangiella traps were significantly divergent between traps of different cruises (*p* < 0.05).

Ninety single cells were isolated by flow cytometry at the Single Cell Genomics Center at the Bigelow Laboratory for Ocean Sciences. Their genomes were Multiple Displacement Amplified (MDA), followed by 16S ribosomal RNA gene amplification and sequencing (Figure S3). Ten cells of the most abundant genera were selected for partial genome sequencing (Table 2). From the 110 m live trap, two cells for Idiomarina and two cells for Kangiella were sequenced, and from the 500 m live trap one cell was chosen for Actinomarina. From the 110 m poisoned trap two cells were chosen for Vibrio, and for the 500 m poisoned trap two cells were chosen for Arcobacter and one cell for the SAR11 clade. All four pairs of SAGs from the same genus were closely related, with identical 16S rRNA gene sequences and high average nucleotide identity (>95%; Figure S5). Interestingly, all four of these genera were highlighted in analyses of microbes associated with export in the upper water column from the Tara expedition, as being important for carbon export (Guidi et al., 2015). The genomes were analyzed for genetic features (Figure 3, Table 2), taxonomic signals (Figures S4–S6) and gene content (Figure 4; Table S2), as well as relative read recruitment from the metagenomes (Figure 5, Figure S7).

### Initial microbial assemblages in the poisoned traps

The poisoned trap metagenomes represented cells on sinking particles that were immediately fixed by the preservative as they entered the trap. We refer material in the posoined traps, consisting of eukaryotes that make up the bulk of the particles and their associated bacteria as the “initial community.” Eukaryotes and associated viruses were more common in the initial community, than in the live traps (Figure 1). Many of the significantly more abundant bacteria in poisoned traps were either associated with eukaryotes (Arcobacter, Ilumatobacter, and Planctomycetes) or potentially with the micro-niches specific to particles (Chroococcales, Sulfurimonas, and Oceanospirillales; Figure 2). The phototroph Cyanothece was also identified, which is known to form aggregates and thus might have contributed to the particle formation. Ammonia oxidizing archaea and bacteria were significantly enriched in this fraction, despite low overall abundance in the traps.

Bacterial genes significantly enriched in the poisoned traps included components for sulfate reduction, cyanobacterial photosynthesis, and carbon metabolism, possibly carbon fixation by the reductive TCA cycle (Table S1c). There were genes for cell signaling, secretion and environmental information processing, and genes for uptake of zinc, manganese, biotin, and oligopeptides. Genes encoded by the Arcobacter and Vibrio SAGs were enriched primarily in the poisoned traps (Figure 4), where Arcobacter—but not Vibrio—were significantly enriched in the metagenomes on a genus marker gene level (Figure 2).

### Arcobacter

Two closely related Arcobacter SAGs were isolated from the 500 m poisoned trap during the second cruise (HD9). Although Arcobacter is best known as an animal pathogen, the genus contains both animal associated and free-living pathogenic and non-pathogenic strains. It is known to form mutualistic relationships with the amoeboflagellate Breviatea (Colomban de Vargas et al., 2015; Hamann et al., 2016). Arcobacter representatives have also been found as part of the biofilm community on sunken wood where they have been suggested to mineralize sulfur and fix nitrogen (Kalenitchenko et al., 2016).

These SAGs cluster together with the marine strain *Arcobacter geojedonensis* (Choe et al., 2015), with a 94% 16S rRNA nucleotide identity to the closest related genome sequenced strain (Figures S4, S5). The highest number of genes were shared with the free-living strain *A. nitrofigilis*, but the level of genome synteny was low, with shared genes dispersed over the genome (Figure S6). The coding densities of the single cell genomes were not as high as those of related strains, which might suggest adaptation to higher nutrient availability (Figure 3, Table 2, Giovannoni et al., 2015). The GC content was relatively low (~30%).

Similar to known strains, the Arcobacter single cell genomes encoded genes for motility and chemotaxis (Table S2). The SAG genes also included the glycerol transporter system *glpPQSTV* as well as the glycerol kinase gene *glpK*, which suggests their ability to use glycerol as a carbon source. The zinc transporter *znuABC* genes were also present in the SAG. The Arcobacter cell encoded several glycoside hydrolases with a putative substrate range that included glucoses, mannoses, xyloses, and peptidoglycan (Table 3, Table S4). In contrast to the other SAGs, and despite their suggested importance in the initial community (Fontanez et al., 2015), the Arcobacter SAGs encoded no glycoside hydrolases specific for chitin, commonly found in eukaryotes including fungi, algae, and crustaceans. This could be a consequence of incomplete representation of the genomes, or it might suggest adaptation to different hosts. Two genes known to be induced in Arcobacter epibionts by the presence of the metazoean *Lenisia limosa* were identified—an Na^+^/H^+^ antiporter of the NhaD family and the 2-oxoglutarate carboxylase *cfiA* (Table S2; Hamann et al., 2016). None of the virulence factors previously described for the pathogen *Arcobacter butzleri* (Douidah et al., 2012) were identified in the SAGs or in assembled contigs of the metagenomes, suggesting that the identified Arcobacter strain is likely a commensialist, rather than a pathogen.

**Table 3:** Glycoside Hydrolases identified in the SAGs annotated in the carbohydrate-active enzymes database (CAZymes).

**Glycoside hydrolase family**	**Substrate**	**SAGs**
GH130	1,6-mannans	Actinomarina, Idiomarina, Kangiella, Vibrio
GH76	1,6-mannans	Idiomarina, Kangiella, SAR11clade, Vibrio
GH109	Acetylgalactosamine	Kangiella, SAR11clade
GH2	Aminosugars	Kangiella, SAR11clade, Vibrio
GH20	Aminosugars	Vibrio
GH5	Cellulose	Actinomarina, Idiomarina
GH9	Cellulose	Vibrio
GH18	Chitin	Actinomarina, Idiomarina, Kangiella, SAR11clade, Vibrio
GH53	Galactans and arabinogalactans	Arcobacter, Idiomarina, Kangiella, Vibrio
GH72	Glucans and galactans	Arcobacter, Idiomarina, Kangiella, SAR11clade, Vibrio
GH16	Glucans and galactans	Arcobacter, Idiomarina, Kangiella, Vibrio
GH3	Glucose, arabinose, xylose	Actinomarina, Idiomarina, Kangiella, Vibrio
GH1	Glucose, galactose	Actinomarina, Arcobacter, Idiomarina, Vibrio
GH84	Hyaluronic Acid	Actinomarina, Idiomarina, Kangiella, SAR11clade, Vibrio
GH99	Mannoses	Actinomarina, Idiomarina, Kangiella, Vibrio
GH92	Mannoses	Arcobacter, Idiomarina, Kangiella, SAR11clade, Vibrio
GH38	Mannoses	Arcobacter, Vibrio
GH47	Mannoses	Kangiella
GH73	Peptidoglycan	Actinomarina, Arcobacter, Idiomarina, SAR11clade, Vibrio
GH23	Peptidoglycan	Arcobacter, Idiomarina, Kangiella, SAR11clade, Vibrio
GH103	Peptidoglycan	Idiomarina, Kangiella, SAR11clade
GH33	Sialic acid	Kangiella, SAR11clade, Vibrio
GH13	Starch	Actinomarina, Idiomarina, Kangiella, Vibrio
GH15	Starch	Idiomarina
GH31	Starch	Kangiella, Vibrio
GH32	Sucrose and fructose containing polymers	Kangiella, SAR11clade, Vibrio
GH107	Unknown	Kangiella
GH24	Unknown	Vibrio
GH10	Xylan	Arcobacter, Idiomarina
GH12	Xyloglucan	Kangiella, Vibrio
GH39	Xylose	Arcobacter, Idiomarina, Kangiella, Vibrio
GH77	α-amylase	Vibrio
GH65	α-glucosidic linkages	Arcobacter, Idiomarina, Vibrio

Arcobacter taxonomic marker genes and reads recruited to the Arcobacter SAGs were almost exclusively found in the 500 m poisoned trap metagenomes, where the SAGs were isolated (Figure 5). This may correspond to the preferred habitat depth of a host eukaryote. A possible candidate for a communalistic partner was identified in two assembled metagenomic contigs containing marker genes related to unicellular protists belonging to the order Collodaria (Biard et al., 2015) that, similar to the Arcobacter SAGs, primarily recruited metagenomic reads from the deep traps (Table S3). This organism has been suggested to be associated with carbon export (Guidi et al., 2015).

### Vibrio

The two Vibrio SAGs clustered with *V. campbellii* affiliated with the *Vibrio harveyi* clade (Figures S4, S5), some strains of which are pathogens of crustacea (Ruwandeepika et al., 2010). The level of genome synteny and shared gene content between the SAGs and reference genomes was high, with the SAGs aligning well to large, discrete parts of both the *V. campbellii* chromosomes and the plasmid NC_009777 (Figure S6). The SAGs were reflective of large genomes typical of copiotrophic organisms, having low coding density and sizable intergenic regions (Figure 3, Table 2). The SAGs encoded peptide transporters (*oppABCD* and *sapACD*) and sugar transporters (*potABCD, proVWX*), as well as transporters of molybdate (modABC), phosphate (pstABCS), and zinc (znuABC). Chitin specific glycoside hydrolases and chitin-binding protein *cbpD* were also present in the Vibrio SAGs, consistent with their potential association with crustaceans. Other glycoside hydrolases with a predicted broad range for other substrates associated with eukaryotes (cellulose, starch, and xyloglucan), bacteria (peptidoglycan), and of general cellular origins (include glucose, mannose, and aminosugars; Table 3, Table S4) were also found. Components of the luminescence quorum sensing machinery (*luxOQRSU*) as well genes homologous to the *rtxA* toxin were present as well. In addition, the gene cysteine synthase (*cysK*), with a suggested role in biofilm formation, was also identified (Table S2; Singh et al., 2015).

**Figure 6:**
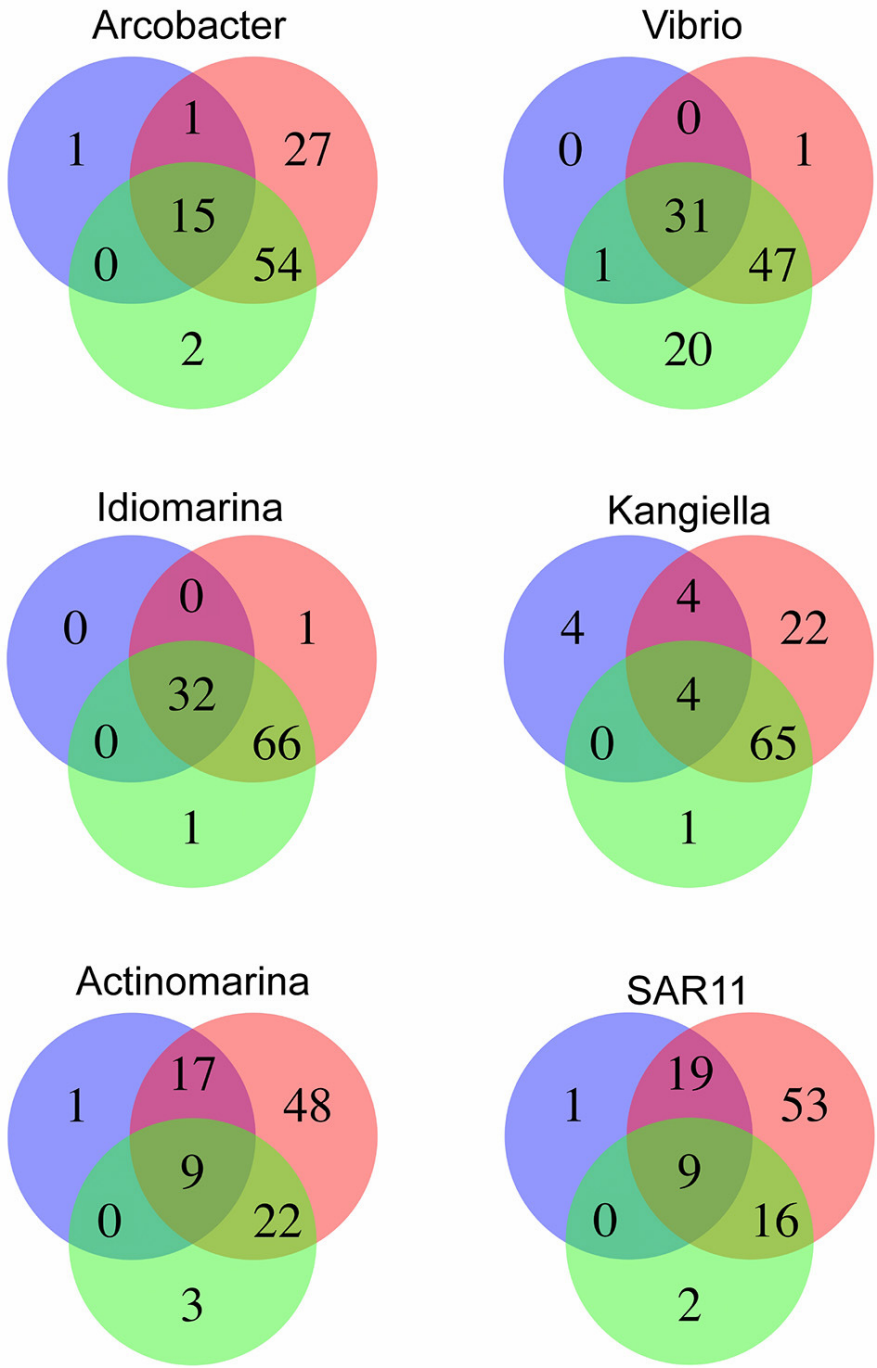
Functional diversity in the traps on KEGG KO level as percentage of total KO diversity for the genus, as predicted by the SAGs (blue), the closest reference genome (red) and the genus specific metagenome from the same HD9 trap as the SAGs were isolated from (green). When combined with the closest reference all SAGs except Vibrio predict the majority of the diversity in the trap, which indicate that the SAG is representative for the functional diversity in the trap. The genus Vibrionales display a substantial functional diversity not predicted by the SAG or closest reference.

The Vibrio SAGs recruited metagenomic reads mostly from the poisoned, shallow traps from which the SAGs were isolated (Figure 5). Although this trend was not statistically significant, it suggested a distribution limited by a eukaryotic host presence, most likely one of the arthropods that were found to be significantly enriched in poisoned traps (Table S1b). This pattern was only seen for reads recruited to the single cell genomes and not in the metagenomes on a taxonomic marker gene level, where Vibrio as a genus was not significantly enriched in any of the sediment traps (Figure 2, Table S1ab). This suggests that different Vibrio strains were present that were adapted to different ecological niches. This observation is obscured when all Vibrio sequence reads are treated cumulatively at the genus level, but can be seen at the higher resolution provided by the single cell genomes. In support of this observation, the diversity of metagenomic functional gene categories for Vibrios in the trap where the SAGs were isolated was larger than what was observed in both Vibrio SAGs along with the *V. campbellii* reference genome (Figure 6). In total, these observations suggested that there were multiple Vibrio strains present in the trap, with different sets of functional genes corresponding to different lifestyles.

### Enriched microbial assemblages in the live traps

The live traps that contained only seawater (without fixative) were enriched in bacteria using nutrients released from the particles during degradation, that we refer to here as the “processed community.” Due to this enrichment, these traps had lower relative numbers of eukaryotes (and their associated viruses) compared to the initial community (Figure 1). The bacteria found here were dominated by known copiotrophic genera including Alteromonas and Idiomarina, their increased numbers resulting presumably from POM or DOM utilization (Figure 2). There were also presumptive hydrocarbon degraders Alcanivorax and Methylophaga that likely were enriched due to reduced carbon compounds associated with the particles. Live traps had fewer uniquely enriched gene suites than poisoned traps, corresponding probably to the lower relative abundance of eukaryotes and their associated microbial assemblages in these samples (Figures 1, 2). This was consistent with the greater amount of specifically enriched bacteria in processed traps, compared to poisoned traps (Table 1, Table S1ab). This likely reflects the smorgasbord of nutrients available for copiotrophic bacteria in the live traps as particles were processed. Enriched bacteria were present at high relative abundance, especially the genus Alteromonas representing nearly one fifth of recovered sequences with similarity to homologs in the RefSeq database (Figure 2), likely reflecting a “bloom” caused by POM and DOM degradation and their accumulation in the live traps. Bacterial genes enriched in live traps included components of pathways for cellular interaction such as two-component systems for signal processing and antibiotics resistance and secretion (Table S1c). There were also genes for the sox sulfur oxidation system (K17224, K17226, K17227) and degradation of amino-acids, nucleotides, and aromatic compounds. Some of the identified iron transport genes were enriched in the live traps (K02014, K16091, K16088; Table S1c), reflecting specialized strategies for iron uptake. This provides further evidence that iron limitation may be strong selective agent for heterotrophic bacteria, as well as oxygenic phototrophs, in the ocean (Boiteau et al., 2016). Genes encoded by the Idiomarina and Kangiella SAGs were enriched primarily in the live traps (Figure 4), where they were significantly enriched also on a genus marker gene level (Figure 2).

### Idiomarina

Idiomarina was first described as a deep-sea bacterium with particle-associated life style and protein metabolism as an important source of nutrients (Donachie et al., 2003; Hou et al., 2004), and representatives have since been found in other environments including shallow water and salterns (e.g., Poddar et al., 2014; León et al., 2015). Isolated *Idiomarina* strains are described as obligately aerobic and heterotrophic, which is generally consistent with the gene content found in the Idiomarina SAGs. The two Idiomarina SAGs had high similarity to described strains (99% 16S rRNA nucleotide identity), with a slightly larger genomic identity to the copiotrophic *I. loihiensis* (Figures S4, S5). There was a high level of genome synteny, with the SAGs corresponding to large, discrete parts of the *I. loihiensis* genome (Figure S6). The genomes were comparably dense, with short intergenic regions (Figure 3, Table 2). The trap-associated Idiomarina SAGs encoded a broad range of glycoside hydrolases, reflecting the DOM released during particle degradation, including putative substrates of both eukaryotic and bacterial origin (Table 3, Table S4). Similar to other Idiomarina, the genomes contained several genes for cell motility and chemotaxis, and also the transporters of phosphate (*pstABCS*) and iron(III) (*afuABC*) as well as the heme exporter *ccmABCD* which allows the cell to regulate the internal iron level (Table S2). The SAGs contained the secretion system *gspCDEFGHIJKL* as well as a Photoactive Yellow Protein (PYP) which has been suggested to be involved in depth adaptive biofilm formation (van Der Horst et al., 2009). However, none of the biofilm forming genes that were identified adjacent to the PYP gene in *I. loihiensis* (Hou et al., 2004) were found in the SAGs, although some of them were present in six assembled contigs of the metagenomes, with highest coverage in the 150 m live trap from HD9 cruise where the Idiomarina SAGs were most common (Table S3). Some Idiomarina genotypes may also be degraders of high molecular weight DOM (McCarren et al., 2010). These findings suggest that Idiomarina in the ocean may associate with sinking particles initially via chemotaxis followed by biofilm formation, and subsequently degrade DOM liberated from POM by other species.

Idiomarina marker genes in the metagenome were significantly enriched in live traps, and in traps from the HD9 cruise. A similar trend was seen by metagenomic reads recruited to the SAGs which were recruited mainly from live traps from most depths (except the deepest sampling point), with a higher fraction of reads recruited from the HD9 metagenomes compared to the HD5 metagenomes (Figure S7), although this trend was not statistically significant (Figure 5). Genes encoded by the Idiomarina SAGs on the other hand, did not show significant differences in abundance between cruises, suggesting a succession between different copiotrophic species with redundant function (Figure S7).

### Kangiella

Kangiella are heterotrophic bacteria first isolated from tidal flats (Yoon et al., 2004). The two Kangiella SAGs form a sister clade to previously known species (Figures S4, S5), with a 96% 16S rRNA nucleotide identity to *K. koreensis* and large, discrete chunks of syntenic genomic regions (Figure S6). The SAGs were less dense than those of *Idiomarina*, with larger intergenic regions with a divergence between the two SAGs most likely explained by different genomic regions being recovered (Figure 3, Table 2). Similar to Idiomarina, the Kangiella SAGs contain multiple glycoside hydrolases with a broad substrate range (Table 3, Table S4). Kangiella are related to the hydrocarbon degrading Alcanivorax, but no such activity has been associated with Kangiella and no genes for hydrocarbon degradation have been identified. Kangiella has been reported to have the ability to degrade lignocellulose (Darjany et al., 2014) as well as algicidal activity (Shi et al., 2013), and there were genes annotated as toxins and antitoxins with potential algicidal activity in the SAGs, including the hemolycin *tlyC*. The Kangiella SAGs encode the peptide transporters *dppACDF*, the phosphate transporter *pstABCS* as well as the iron (III) transport protein *afuB* and the heme exporter *ccmABCD*. There were no genes for motility, either in the SAGs or in the genomes of related strains (Table S2), which is consistent with the non-motility of all known isolated Kangiella strains and the lack of motility genes in the reference genome (Han et al., 2009).

The Kangiella SAGs recruited metagenomic reads from most depths except the deepest sampling point, mostly from live but also from the 150 m poisoned trap (Figure 5). The Kangiella SAGs recruited significantly more reads from the HD9 than the HD5 traps. A similar pattern was seen in the metagenome where Kangiella taxonomic marker genes were significantly enriched in HD9 traps and live traps (Figure S2). The genes encoded by the Kangiella SAGs on an orthology level (KEGG), on the other hand are present in the metagenomes for both cruises with no significantly difference in relative abundance (Figure S8, Table S1c). This suggests a succession between different species with redundant function between cruises, similar to what is hypothesized for Idiomarina.

These findings suggest a model for Kangiella's active role in particle degradation. The non-motile bacteria may be brought to the particles or hosts by Brownian motion and random encounter, and associate with them either as commensialists, or potentially as pathogens. They might then remain attached to sinking particles, accounting for their presence in both live and poisoned traps. As the particles degrade, the Kangiella might then proliferate growing on POM or released DOM.

### Planktonic microbial “bycatch” in the sediment traps

Two of the SAGs (Actinomarina, SAR11 clade) had small predicted genome sizes and low abundance in the metagenome, which suggest planktonic life styles rather than direct association with the particles, and therefore their presence in the traps are thought to be caused by them being caught in the downdraft movement of the particles. Both these SAGs have small, compact genomes which would be consistent with an oligotrophic rather than copiotrophic life style (Figure 3), although they did both contain multiple glycoside hydrolases which suggest at least partial adaption to the DOM released by degrading particles (Table 3, Table S4).

### Actinomarina

The Actinomarina SAG was most closely related to single cell genomes isolated from marine environments with 99% 16S rRNA nucleotide identity (Figures S4, S5; Ghai et al., 2013), but no related strain has been cultivated nor complete reference genome yet published. The Actinomarina SAG coding density was high (Figure 3, Table 2) and included the amino acid transporter *livFGHKM*. The SAG also encoded a proteorhodopsin (PR) belonging to the MAC-cluster of previously described Actinomarina PRs, containing signature residues that suggest adaption to green light (Figure S9, Table S2, Fuhrman et al., 2008). The Actinomarina SAG recruited few reads in the metagenome but was slightly more common in deep traps (Figure 5).

### SAR11 clade

The SAR11 clade SAG that was sequenced shared high nucleotide identity with previously sequenced isolates (99% 16S rRNA nucleotide identity). The genome coding density was high (Figure 3, Table 2) and, similar to *Actinomarina*, encoded the *livFGHKM* amino acid transporter. The genome also contained genes for the sarcosine oxidase *soxABD* (Table S2), which has been noted to be upregulated under nitrogen limited conditions in related organisms (Smith et al., 2013). The SAG recruited few reads from the metagenomes, but appeared slightly more common in the deep traps (Figure 5).

### Single amplified genomes as models

The SAGs can serve as models of potential successional strategies that occur during sinking particle degradation. To estimate how representative they might be for trap microorganisms, the gene content of the SAGs was compared to that of the corresponding metagenomes. Genomic regions not recovered in the single cell genomes were compensated for by also comparing SAGs to their closest relative with a fully sequenced reference genome. The full genetic diversity of organisms that belong to the same genera as the SAGs in the samples were then estimated by isolating genera-specific reads from the metagenomes of the trap corresponding to the SAGs. The gene content of KEGG orthologous groups were predicted from these three datasets and compared (Figure 6).

Except for the Vibrio SAGs, most of the genes from the metagenome were also encoded by the SAGs, which suggest that there is low diversity of the genus in the trap and that the SAGs were reasonably representative of members of the trap bacterial community. The Vibrio genus on the other hand, displayed a greater diversity in the trap metagenomes than what was represented by the SAGs. The pathogenic life style suggested for the Vibrio SAGs might therefore not be the only ecological adaption for Vibrio in the traps, and further sampling would be required to obtain a larger genomic representation of Vibrio associated with sinking particles.

### Depth distributions of microbes captured by the sediment traps

When samples from different depths were compared, there were four genera of alpha and gammaproteobacteria significantly enriched in shallow compared to deep traps—Acinetobacter, Ferrimonas, Maricaulis, and Pseudoalteromonas. In deep traps, eight epsilonproteobacterial genera (mainly Campylobacterales) were enriched (Table S1ab). There was an overlap between groups enriched in deep and poisoned traps, contrasted to shallow and live, as well as an overlap between shallow and live traps (Figure S2). Interestingly, no such overlap was seen between groups enriched in deep and live traps, or shallow, and poisoned. Similarly, the deep, poisoned traps were enriched in Epsilonproteobacteria, mainly Campylobacterales (Arcobacter, Nitratiruptor, Sulfuricurvum, Sulfurimonas, and Sulfurospirillum) while the shallow, live traps were enriched in Alphaproteobacteria and Gammaproteobacteria (Acinetobacter, Ferrimonas, Maricaulis, and Pseudoalteromonas), suggesting a shift in either particle composition or bacterial recruitment as the particles move downwards in the water column. Only a small number of genes were significantly divergent in abundance between shallow and deep traps, including genes for aromatic degradation (K01821) and carbohydrate and nucleotide metabolism (K01846, K02293, K00619, K18284)—all of them enriched in deep traps (>300 m; Table S1c).

### Difference between cruises

Only five bacterial genera displayed differential representation on particles collected in the two different cruises. Four of them (Brevundimonas, Herbaspirillum, Idiomarina, and Kangiella; Table S1ab) were significantly enriched in the live traps as well as during the later cruise (HD9). In contrast, 16 of 18 Eukaryotic genera (Figure S2, Table S1ab), significantly enriched in the later cruise (HD9), were enriched in poisoned traps. This suggests a relatively stable bacterial system during the time frame of this study even as the eukaryotic community itself shifted.

## Discussion

Particles that sink to the dark ocean appear to be predominantly composed of eukaryotic biomass (Simon et al., 2002; Fontanez et al., 2015; Guidi et al., 2015). This may include the presence of fecal pellets, diatom aggregates, a variety of protists and their associated microflora, as well as non-organic components. Most recently, the Tara expedition has highlighted the association of both prokaryotes and viruses, as well as previously overlooked eukaryotic groups including Rhizaria, with carbon export and potentially sinking particles (Fontanez et al., 2015; Guidi et al., 2015).

Microbes attached to sinking particles in the upper water column have been suggested to be primarily recruited in the photic zone, so that particles collected at greater depths would be predicted to harbor microbes from photic zone, rather than in surrounding deep water. The recruitment of metagenomic reads to the SAGs appeared to be in general agreement at least for these upper mesopelagic samples, as we observed enrichment of specific microbial communities in shallow live traps and deep poisoned traps, but no enrichment of specific groups in shallow poisoned traps or deep live traps.

In a simplified model the microbial assemblages on sinking particles may be affected mainly by recruitment in shallow water, and by microbes associated with depth-specific eukaryotes. The initial microbial assemblage of the shallow poisoned traps consisted of eukaryotic cells with their associated pathogens, predators, commensalists, and saprophytes (represented by Vibrio and Kangiella), as well as recruited motile copiotrophs (represented by Idiomarina). Correspondingly, the initial assemblage of the deep poisoned traps consisted of the same copiotrophs—recruited from the photic zone—but also along with a set of unique depth-specific eukaryotic associated microbes (represented by Arcobacter).

The above model may explain why no bacterial groups were specifically enriched in the live, deep traps. The particle-enriched assemblage in these traps was expected to be seeded by copiotrophs recruited in shallow depths, and therefore not be unique for either depth. In addition, bacteria enriched in the deep, poisoned trap appear associated with eukaryotes specifically adapted to these depths, as exemplified by Arcobacter, rather than having been transported from the photic zone.

Particles are hotspots for microbial life in the deep ocean, and the lifestyles represented by the trap associated microbial assemblage genes and genomes demonstrate some of their important adaptations to this environment. The Arcobacter and Vibrio SAG data seemed to indicate they were eukaryotic-associated and adapted to specific depths, most likely dictated by the preference of their host(s). Arcobacter was apparently a commensalist, and Vibrio either a commensalist or possibly a pathogen, with genes encoding both toxins, quorum sensing and chitinase. Both strains were present in both cruises in relatively high numbers in the initial community, but were rapidly outcompeted by generalist copiotrophs as the particles were processed. *Idiomarina* spp., appeared as copiotrophs present in live traps at several depths of the water column, potentially migrating to particles via chemotaxis. In contrast, *Kangiella* spp., were presumably non-motile and seemed to be present in both the initial as well as processed assemblages. They may possibly act as both as a non-specific pathogens (given their encoded toxins), and as saprophytes. The Actinomarina and SAR11 clade SAGs with small, dense genomes, were not significantly enriched by the presence of the particles generally consistent with their presumptive planktonic lifestyles.

The combination of SAG and metagenome data we used proved useful for gaining a deeper perspective on microorganisms inhabiting and degrading sinking particles. The distributions and gene contents of the Arcobacter, Idiomarina, and Kangiella SAGs suggested some of their functional roles on particulates. Our findings corroborate and extend an earlier study, where the importance of eukaryotes for determining the initial microbial community of sinking particles was proposed (Fontanez et al., 2015). Specific ecological strategies for particle processing were indicated by single cell genomes of Arcobacter, Vibrio, Idiomarina, and Kangiella, furthering our knowledge of bacterial processes and sucession on sinking particles in the open ocean.

## Author contributions

EP, KF, and ED designed research. EP sequenced and analyzed single cell genomes and analyzed metagenomes. KF designed sediment traps and sequenced metagenomes as well as performed initial metagenomic analysis. EP, KF, and ED wrote the paper.

### Conflict of interest statement

The authors declare that the research was conducted in the absence of any commercial or financial relationships that could be construed as a potential conflict of interest.
