# Supplementary material for: Bacterial Succession on Sinking Particles in the Ocean's Interior
Source: Front Microbiol. 2017 Nov 24;8:2269. doi: 10.3389/fmicb.2017.02269 (PMC5706468; doi:10.3389/fmicb.2017.02269)
Supplement: Supplementary file 1 [file DataSheet1.pdf]

# Supplemental Information

## Bacterial succession on sinking particles in the ocean's interior

**Erik A. Pelve, Kristina M. Fontanez and Edward F. DeLong**

**Table S5.** Metagenome overview

| Sample | Cruise | Treatment | Depth | Refseq_hits | Silva_hits | KEGG_hits | Fractions |
|---|---|---|---|---|---|---|---|
| HD5_150P | HD5 | P | 150m (SHALLOW) | 643371 | 34185 | 391351 | 0.22-335 µm |
| HD5_150L | HD5 | L | 150m (SHALLOW) | 1795319 | 13129 | 1027202 | 0.22-335 µm |
| HD5_200P | HD5 | P | 200m (SHALLOW) | 213836 | 7466 | 121605 | 0.22-335 µm |
| HD5_200L | HD5 | L | 200m (SHALLOW) | 429533 | 3101 | 242724 | 0.22-335 µm |
| HD5_300P | HD5 | P | 300m (DEEP) | 54517 | 2445 | 30843 | 0.22-335 µm |
| HD5_300L | HD5 | L | 300m (DEEP) | 1026497 | 7839 | 634097 | 0.22-335 µm |
| HD5_500P | HD5 | P | 500m (DEEP) | 177333 | 6924 | 107745 | 0.22-335 µm |
| HD5_500L | HD5 | L | 500m (DEEP) | 549597 | 7356 | 348376 | 0.22-335 µm |
| HD9_110P | HD9 | P | 110m (SHALLOW) | 2068117 | 40988 | 1244209 | 0.22, 5 µm |
| HD9_110L | HD9 | L | 110m (SHALLOW) | 1067596 | 7590 | 628606 | 0.22, 5 µm |
| HD9_150P | HD9 | P | 150m (SHALLOW) | 281431 | 6126 | 168068 | 0.22, 5 µm |
| HD9_150L | HD9 | L | 150m (SHALLOW) | 1209680 | 10752 | 758264 | 0.22, 5 µm |
| HD9_200P | HD9 | P | 200m (SHALLOW) | 785458 | 9427 | 459348 | 0.22, 5 µm |
| HD9_200L | HD9 | L | 200m (SHALLOW) | 3524662 | 27599 | 2125461 | 0.22, 5 µm |
| HD9_300P | HD9 | P | 300m (DEEP) | 34080 | 1064 | 17930 | 5 µm |
| HD9_300L | HD9 | L | 300m (DEEP) | 1634965 | 10125 | 979106 | 0.22, 5 µm |
| HD9_500P | HD9 | P | 500m (DEEP) | 68561 | 2460 | 37977 | 0.22, 5 µm |
| HD9_500L | HD9 | L | 500m (DEEP) | 1887713 | 21441 | 1208975 | 0.22, 5 µm |

# PCA of ordinations

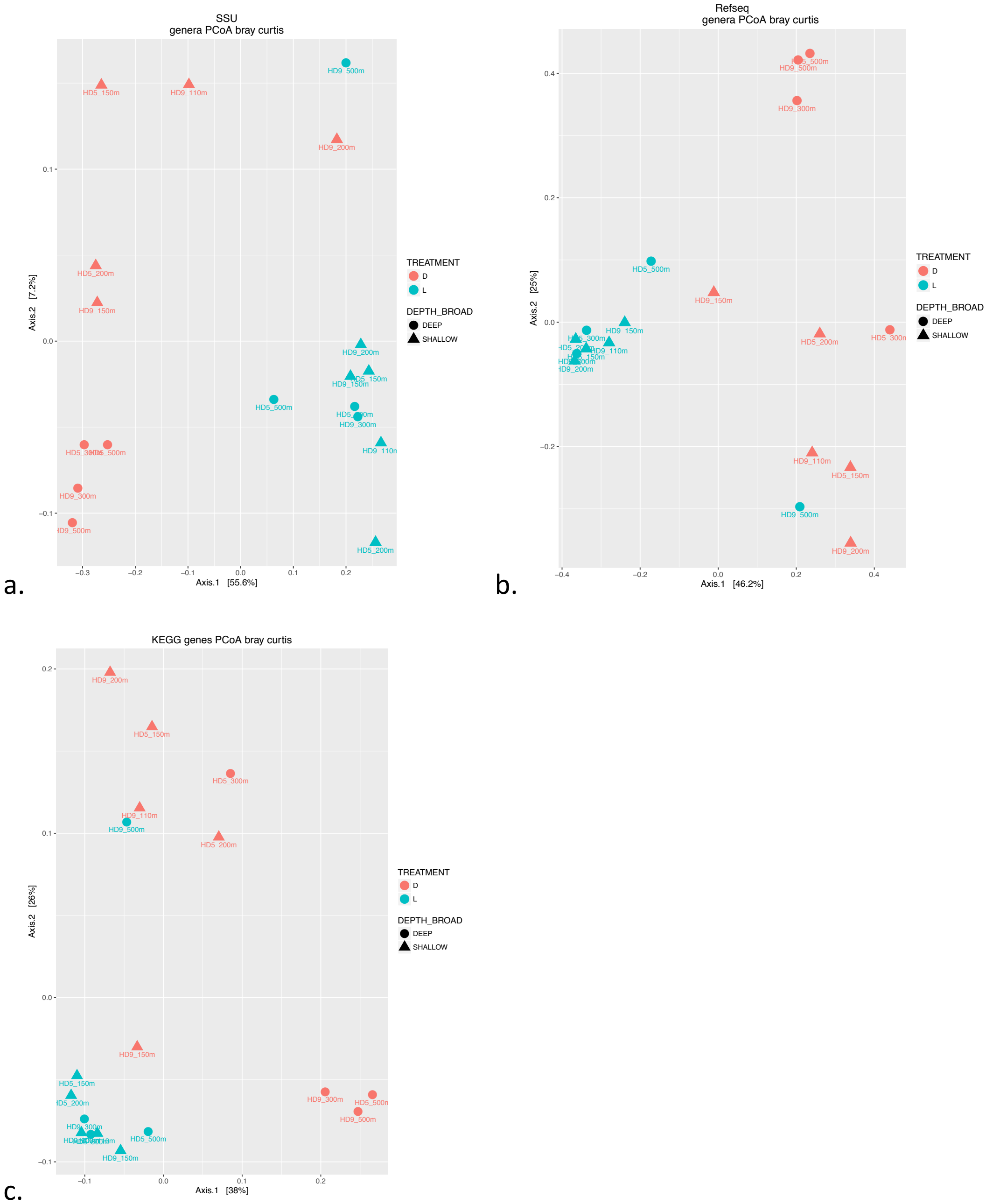


**Figure S1.** PCA for bray curtis ordinations of a) Silva, b) Refseq and c) KEGG database hits.

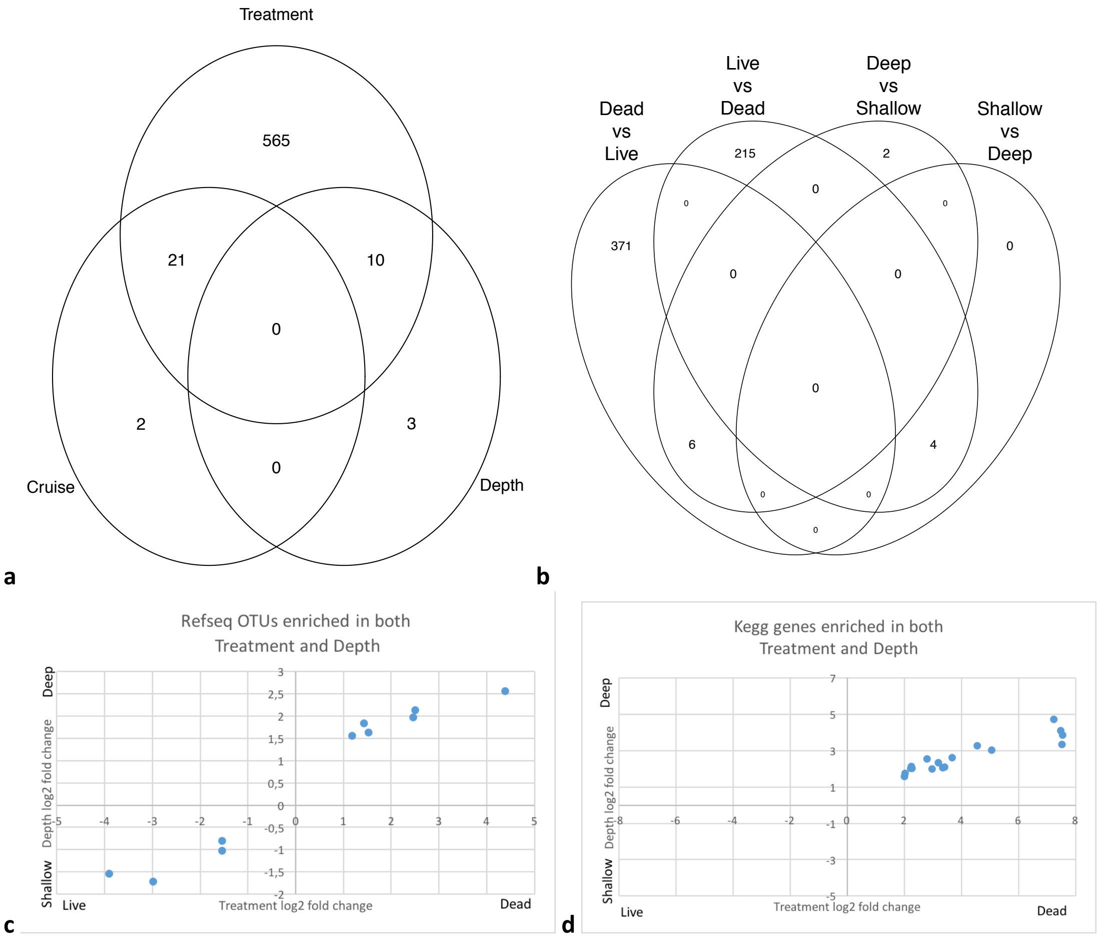


**Figure S2.** OTUs enriched in multiple dimensions. a) Refseq comparison between enriched OTUs in all dimensions. b) Refseq comparison between Treatment and Depth. c) Refseq OTUs enriched in both Treatment and Depth. d) KEGG OTUs enriched in both Treatment and Depth. e). Refseq distribution of individual OTUs enriched in both Dead and Deep traps. F) Refseq distributions of individual OTUs enriched in both Live and Shallow traps. G). KEGG distripution of individual genes enriched in both Dead and Deep traps.

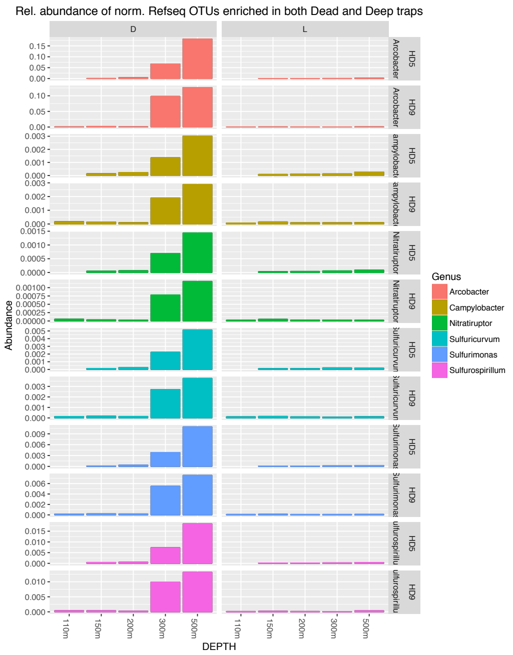
Rel. abundance of norm. Refseq OTUs enriched in both Dead and Deep traps
D
L
Abundance
DEPTH
110m
150m
200m
300m
500m
Genus
Arcobacter
Campylobacter
Nitratiruptor
Sulfuricurvum
Sulfurimonas
Sulfurospirillum

e

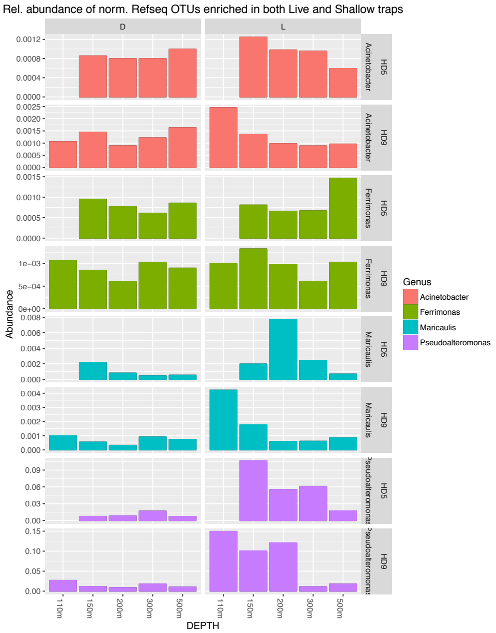
Rel. abundance of norm. Refseq OTUs enriched in both Live and Shallow traps
D
L
Abundance
DEPTH
110m
150m
200m
300m
500m
Genus
Acinetobacter
Ferrimonas
Maricaulis
Pseudoalteromonas

f

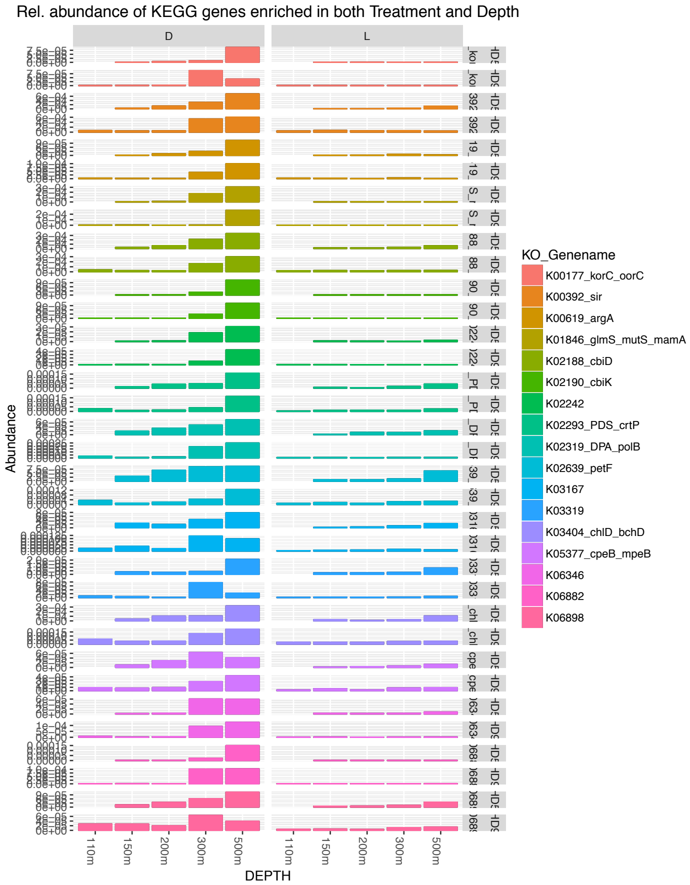
Rel. abundance of KEGG genes enriched in both Treatment and Depth
D
L
Abundance
DEPTH
110m
150m
200m
300m
500m
KO_Genename
K00177_korC_oorC
K00392_sir
K00619_argA
K01846_glmS_mutS_mamA
K02188_cbiD
K02190_cbiK
K02242
K02293_PDS_crtP
K02319_DPA_polB
K02639_petF
K03167
K03319
K03404_chlD_bchD
K05377_cpeB_mpeB
K06346
K06882
K06898

g

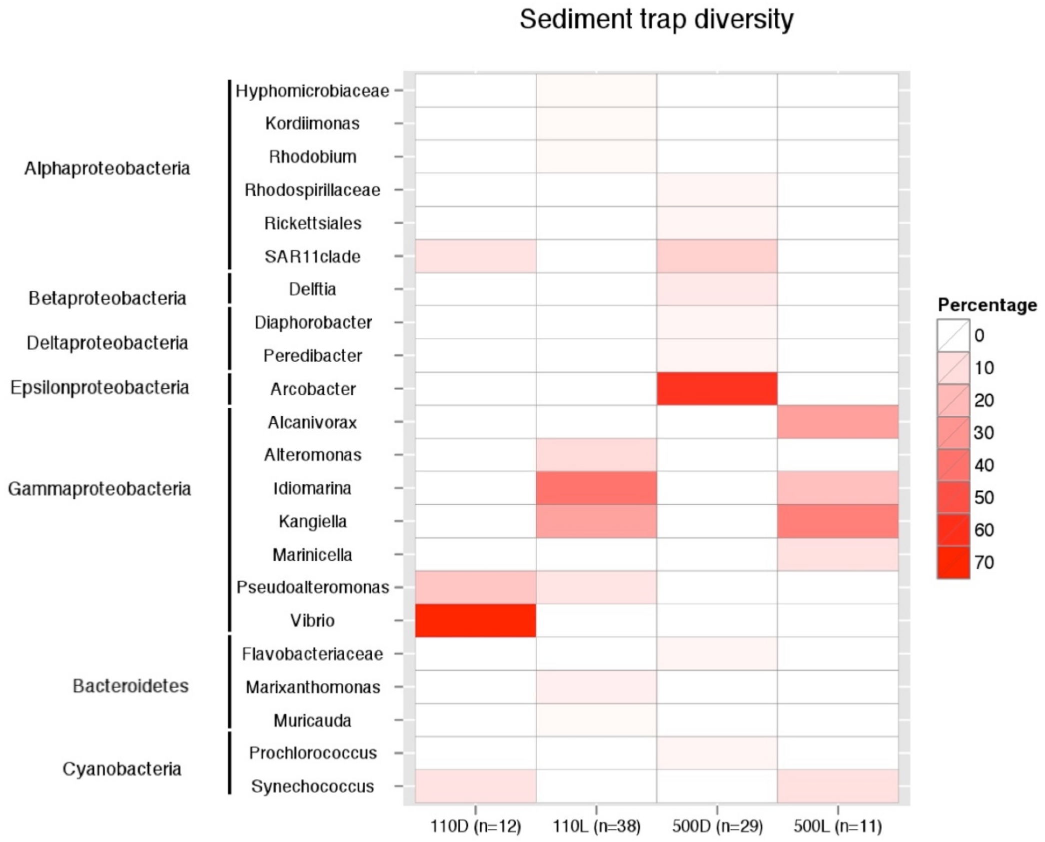


**Figure S3.** Genera distribution of HD9 trap SAG SSU amplicons, mapped to the SILVA 115 database by LAST. Cutoff: lastscore 50, e value 0.01.

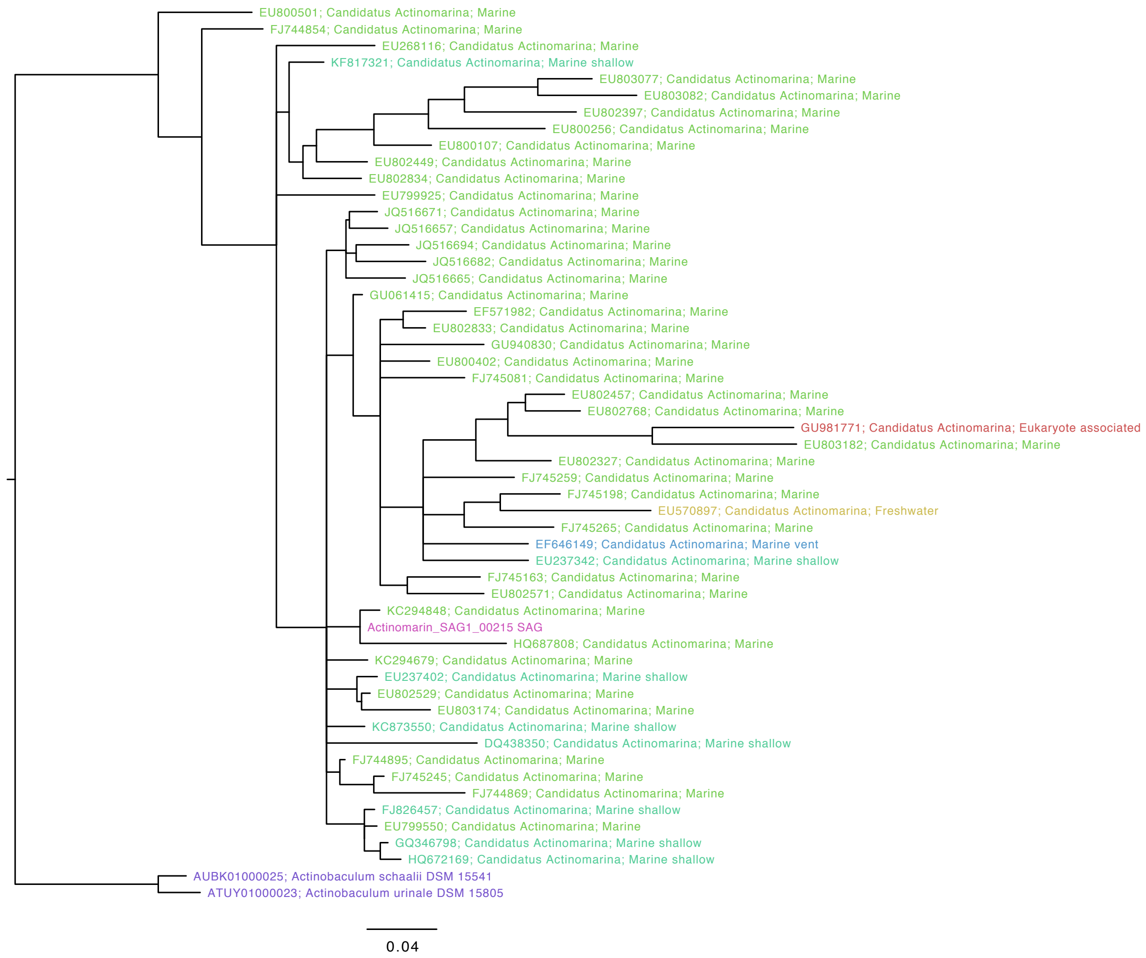


**Figure S4a.** Actinomarina SSU tree (1000 bp size limit except SAGs, only sequences with annotated origin; usearch 95%, linsi (auto); BMGE (DNAPAM1:2); IQ-tree (Model TIM3e+I+G4; fast bootstrapping, 1000 replicates; Only branches with >50% bootstrap support represented)).

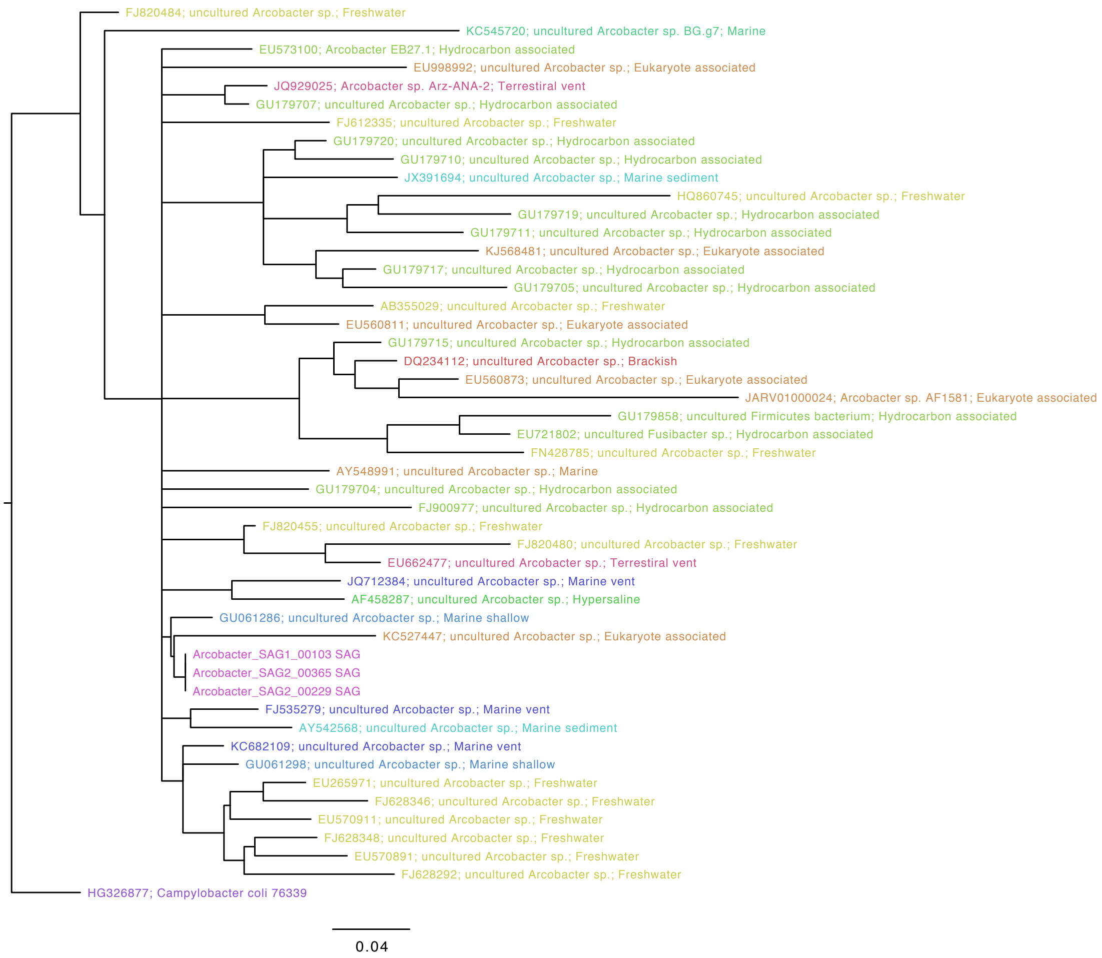


**Figure S4b.** Arcobacter SSU tree (1000 bp size limit except SAGs, only sequences with annotated origin; usearch 95%;, linsi (auto); BMGE (DNAPAM1:2); IQ-tree (Model TIM+I+G4; fast bootstrapping, 1000 replicates; Only branches with >50% bootstrap support represented)).

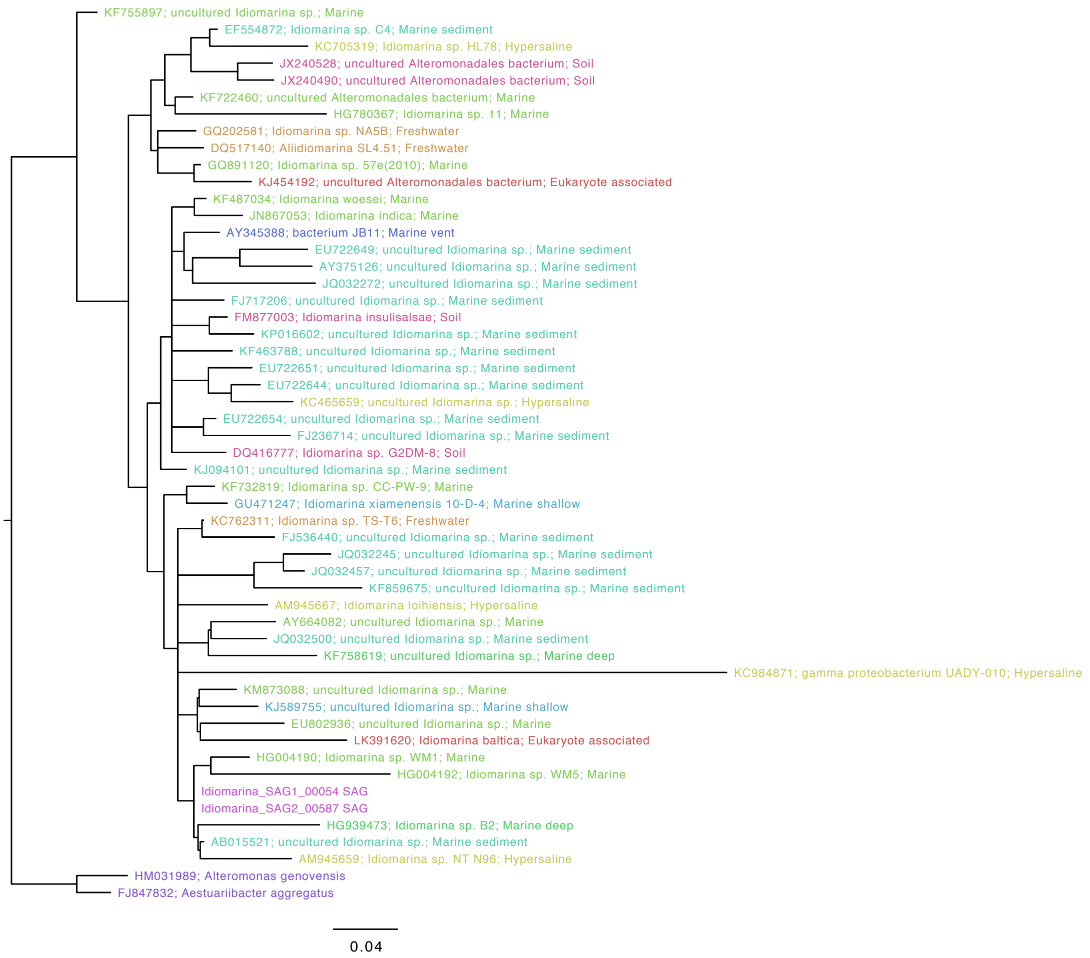


**Figure S4c.** Idiomarina SSU tree (1000 bp size limit except SAGs, only sequences with annotated origin; usearch 95%;, linsi (auto); BMGE (DNAPAM1:2); IQ-tree (Model TIM3+G4; fast bootstrapping, 1000 replicates; Only branches with >50% bootstrap support represented)).

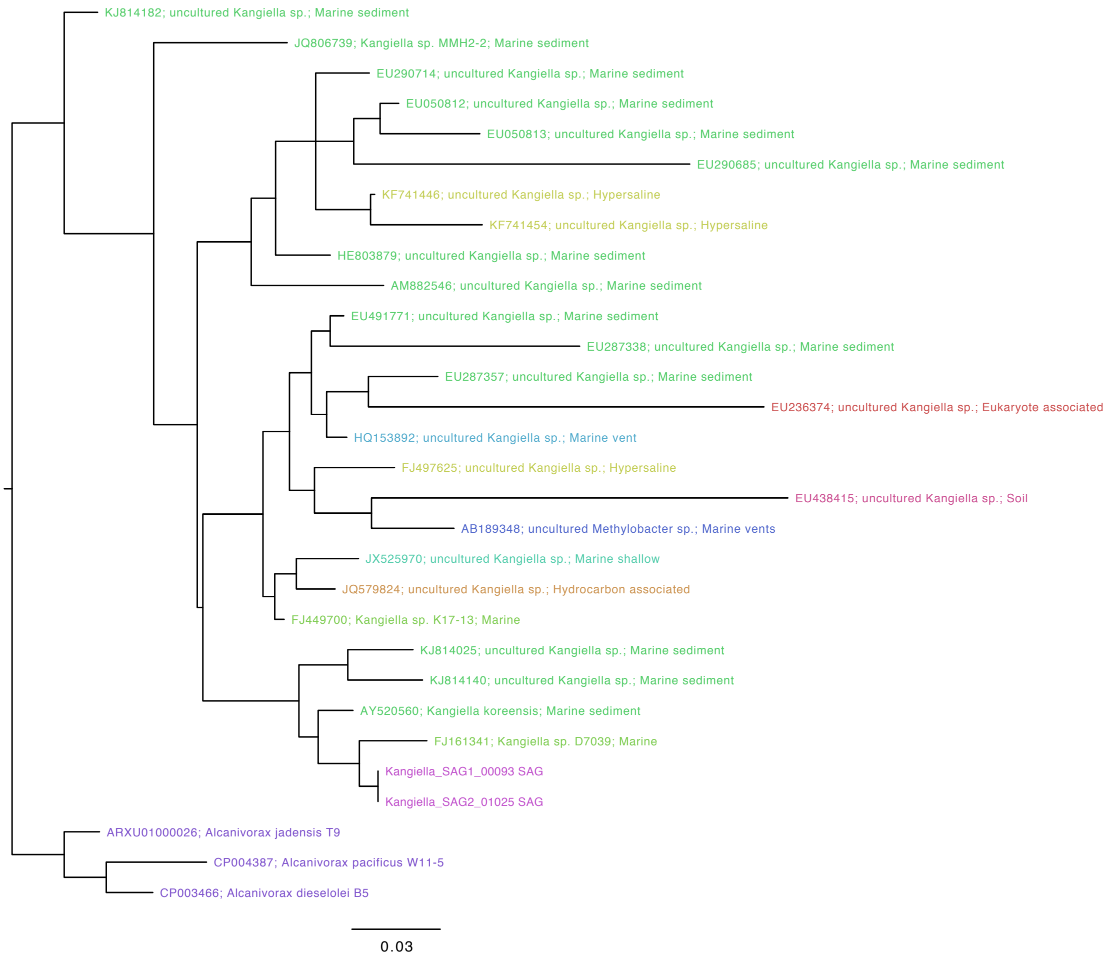


**Figure S4d.** Kangiella SSU tree (1000 bp size limit except SAGs, only sequences with annotated origin; usearch 95%;, linsi (auto); BMGE (DNAPAM1:2); IQ-tree (Model TIM3+I+G4; fast bootstrapping, 1000 replicates; Only branches with >50% bootstrap support represented)).

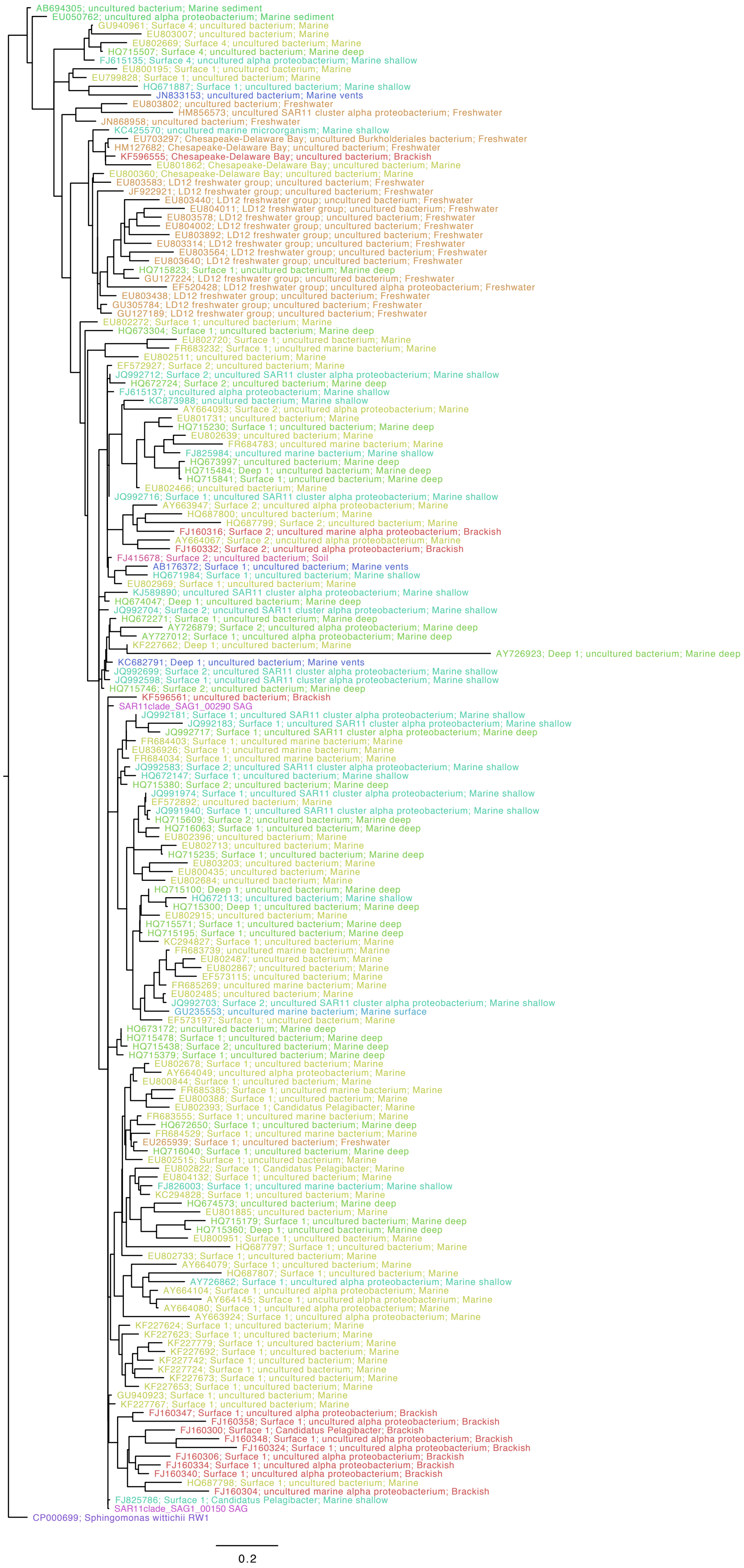

AB694305; uncultured bacterium; Marine sediment
EU050762; uncultured alpha proteobacterium; Marine sediment
GU940961; Surface 4; uncultured bacterium; Marine
EU803007; uncultured bacterium; Marine
EU802669; Surface 4; uncultured bacterium; Marine
HQ715507; Surface 4; uncultured bacterium; Marine deep
FJ615135; Surface 4; uncultured alpha proteobacterium; Marine shallow
EU800195; Surface 1; uncultured bacterium; Marine
EU799828; Surface 1; uncultured bacterium; Marine
HQ671887; Surface 1; uncultured bacterium; Marine shallow
JN833153; uncultured bacterium; Marine vents
EU803802; uncultured bacterium; Freshwater
HM856573; uncultured SAR11 cluster alpha proteobacterium; Freshwater
JN868958; uncultured bacterium; Freshwater
KC425570; uncultured marine microorganism; Marine shallow
EU703297; Chesapeake-Delaware Bay; uncultured Burkholderiales bacterium; Freshwater
HM127682; Chesapeake-Delaware Bay; uncultured bacterium; Freshwater
KF596555; Chesapeake-Delaware Bay; uncultured bacterium; Brackish
EU801862; Chesapeake-Delaware Bay; uncultured bacterium; Marine
EU800360; Chesapeake-Delaware Bay; uncultured bacterium; Marine
EU803583; LD12 freshwater group; uncultured bacterium; Freshwater
JF922921; LD12 freshwater group; uncultured bacterium; Freshwater
EU803440; LD12 freshwater group; uncultured bacterium; Freshwater
EU804011; LD12 freshwater group; uncultured bacterium; Freshwater
EU803578; LD12 freshwater group; uncultured bacterium; Freshwater
EU804002; LD12 freshwater group; uncultured bacterium; Freshwater
EU803892; LD12 freshwater group; uncultured bacterium; Freshwater
EU803314; LD12 freshwater group; uncultured bacterium; Freshwater
EU803564; LD12 freshwater group; uncultured bacterium; Freshwater
EU803640; LD12 freshwater group; uncultured bacterium; Freshwater
HQ715823; Surface 1; uncultured bacterium; Marine deep
GU127224; LD12 freshwater group; uncultured bacterium; Freshwater
EF520428; LD12 freshwater group; uncultured alpha proteobacterium; Freshwater
EU803438; LD12 freshwater group; uncultured bacterium; Freshwater
GU305784; LD12 freshwater group; uncultured bacterium; Freshwater
GU127189; LD12 freshwater group; uncultured bacterium; Freshwater
EU802272; Surface 1; uncultured bacterium; Marine
HQ673304; Surface 1; uncultured bacterium; Marine deep
EU802720; Surface 1; uncultured bacterium; Marine
FR683232; Surface 1; uncultured marine bacterium; Marine
EU802511; uncultured bacterium; Marine
EF572927; Surface 2; uncultured bacterium; Marine
JQ992712; Surface 2; uncultured SAR11 cluster alpha proteobacterium; Marine shallow
HQ672724; Surface 2; uncultured bacterium; Marine deep
FJ615137; uncultured alpha proteobacterium; Marine shallow
KC873988; uncultured bacterium; Marine shallow
AY664093; Surface 2; uncultured alpha proteobacterium; Marine
EU801731; uncultured bacterium; Marine
HQ715230; Surface 1; uncultured bacterium; Marine deep
EU802639; uncultured bacterium; Marine
FR684783; uncultured marine bacterium; Marine
FJ825984; uncultured marine bacterium; Marine shallow
HQ673997; uncultured bacterium; Marine deep
HQ715484; Deep 1; uncultured bacterium; Marine deep
HQ715841; Surface 1; uncultured bacterium; Marine deep
EU802466; uncultured bacterium; Marine
JQ992716; Surface 1; uncultured SAR11 cluster alpha proteobacterium; Marine shallow
AY663947; Surface 2; uncultured alpha proteobacterium; Marine
HQ687800; uncultured bacterium; Marine
HQ687799; Surface 2; uncultured bacterium; Marine
FJ160316; Surface 2; uncultured marine alpha proteobacterium; Brackish
AY664067; Surface 2; uncultured alpha proteobacterium; Marine
FJ160332; Surface 2; uncultured alpha proteobacterium; Brackish
FJ415678; Surface 2; uncultured bacterium; Soil
AB176372; Surface 1; uncultured bacterium; Marine vents
HQ671984; Surface 1; uncultured bacterium; Marine shallow
EU802969; Surface 1; uncultured bacterium; Marine
KJ589890; uncultured SAR11 cluster alpha proteobacterium; Marine shallow
HQ674047; Deep 1; uncultured bacterium; Marine deep
JQ992704; Surface 2; uncultured SAR11 cluster alpha proteobacterium; Marine shallow
HQ672271; Surface 1; uncultured bacterium; Marine deep
AY726879; Surface 2; uncultured alpha proteobacterium; Marine deep
AY727012; Surface 1; uncultured alpha proteobacterium; Marine deep
KF227662; Deep 1; uncultured bacterium; Marine
AY726923; Deep 1; uncultured bacterium; Marine deep
KC682791; Deep 1; uncultured bacterium; Marine vents
JQ992699; Surface 2; uncultured SAR11 cluster alpha proteobacterium; Marine shallow
JQ992598; Surface 1; uncultured SAR11 cluster alpha proteobacterium; Marine shallow
HQ715746; Surface 2; uncultured bacterium; Marine deep
KF596561; uncultured bacterium; Brackish
SAR11clade_SAG1_00290 SAG
JQ992181; Surface 1; uncultured SAR11 cluster alpha proteobacterium; Marine shallow
JQ992183; Surface 1; uncultured SAR11 cluster alpha proteobacterium; Marine shallow
JQ992717; Surface 1; uncultured SAR11 cluster alpha proteobacterium; Marine deep
FR684403; Surface 1; uncultured marine bacterium; Marine
EU836926; Surface 1; uncultured marine bacterium; Marine
FR684034; Surface 1; uncultured marine bacterium; Marine
JQ992583; Surface 2; uncultured SAR11 cluster alpha proteobacterium; Marine shallow
HQ672147; Surface 1; uncultured bacterium; Marine shallow
HQ715380; Surface 2; uncultured bacterium; Marine deep
JQ991974; Surface 1; uncultured SAR11 cluster alpha proteobacterium; Marine shallow
EF572892; uncultured bacterium; Marine
JQ991940; Surface 1; uncultured SAR11 cluster alpha proteobacterium; Marine shallow
HQ715609; Surface 2; uncultured bacterium; Marine deep
HQ716063; Surface 1; uncultured bacterium; Marine deep
EU802396; uncultured bacterium; Marine
EU802713; uncultured bacterium; Marine
HQ715235; Surface 1; uncultured bacterium; Marine deep
EU803203; uncultured bacterium; Marine
EU800435; uncultured bacterium; Marine
EU802684; uncultured bacterium; Marine
HQ715100; Deep 1; uncultured bacterium; Marine deep
HQ672113; uncultured bacterium; Marine shallow
HQ715300; Deep 1; uncultured bacterium; Marine deep
EU802915; uncultured bacterium; Marine
HQ715571; Surface 1; uncultured bacterium; Marine deep
HQ715195; Surface 1; uncultured bacterium; Marine deep
KC294827; Surface 1; uncultured bacterium; Marine
FR683739; uncultured marine bacterium; Marine
EU802487; uncultured bacterium; Marine
EU802867; uncultured bacterium; Marine
EF573115; uncultured bacterium; Marine
FR685269; uncultured marine bacterium; Marine
EU802485; uncultured bacterium; Marine
JQ992703; Surface 2; uncultured SAR11 cluster alpha proteobacterium; Marine shallow
GU235553; uncultured marine bacterium; Marine surface
EF573197; Surface 1; uncultured bacterium; Marine
HQ673172; uncultured bacterium; Marine deep
HQ715478; Surface 1; uncultured bacterium; Marine deep
HQ715438; Surface 2; uncultured bacterium; Marine deep
HQ715379; Surface 1; uncultured bacterium; Marine deep
EU802678; Surface 1; uncultured bacterium; Marine
AY664049; uncultured alpha proteobacterium; Marine
EU800844; Surface 1; uncultured bacterium; Marine
FR685385; Surface 1; uncultured marine bacterium; Marine
EU800388; Surface 1; uncultured bacterium; Marine
EU802393; Surface 1; Candidatus Pelagibacter; Marine
FR683555; Surface 1; uncultured marine bacterium; Marine
HQ672650; Surface 1; uncultured bacterium; Marine deep
FR684529; Surface 1; uncultured marine bacterium; Marine
EU265939; Surface 1; uncultured bacterium; Freshwater
HQ716040; Surface 1; uncultured bacterium; Marine deep
EU802515; Surface 1; uncultured bacterium; Marine
EU802822; Surface 1; Candidatus Pelagibacter; Marine
EU804132; Surface 1; uncultured bacterium; Marine
FJ826003; Surface 1; uncultured marine bacterium; Marine shallow
KC294828; Surface 1; uncultured bacterium; Marine
HQ674573; uncultured bacterium; Marine deep
EU801885; uncultured bacterium; Marine
HQ715179; Surface 1; uncultured bacterium; Marine deep
HQ715360; Deep 1; uncultured bacterium; Marine deep
EU800951; Surface 1; uncultured bacterium; Marine
HQ687797; Surface 1; uncultured bacterium; Marine
EU802733; Surface 1; uncultured bacterium; Marine
AY664079; Surface 1; uncultured bacterium; Marine
HQ687807; Surface 1; uncultured bacterium; Marine
AY726862; Surface 1; uncultured alpha proteobacterium; Marine shallow
AY664104; Surface 1; uncultured alpha proteobacterium; Marine
AY664145; Surface 1; uncultured alpha proteobacterium; Marine
AY664080; Surface 1; uncultured alpha proteobacterium; Marine
AY663924; Surface 1; uncultured alpha proteobacterium; Marine
KF227624; Surface 1; uncultured bacterium; Marine
KF227623; Surface 1; uncultured bacterium; Marine
KF227779; Surface 1; uncultured bacterium; Marine
KF227692; Surface 1; uncultured bacterium; Marine
KF227742; Surface 1; uncultured bacterium; Marine
KF227724; Surface 1; uncultured bacterium; Marine
KF227673; Surface 1; uncultured bacterium; Marine
KF227653; Surface 1; uncultured bacterium; Marine
GU940923; Surface 1; uncultured bacterium; Marine
KF227767; Surface 1; uncultured bacterium; Marine
FJ160347; Surface 1; uncultured alpha proteobacterium; Brackish
FJ160358; Surface 1; uncultured alpha proteobacterium; Brackish
FJ160300; Surface 1; Candidatus Pelagibacter; Brackish
FJ160348; Surface 1; uncultured alpha proteobacterium; Brackish
FJ160324; Surface 1; uncultured alpha proteobacterium; Brackish
FJ160306; Surface 1; uncultured alpha proteobacterium; Brackish
FJ160334; Surface 1; uncultured alpha proteobacterium; Brackish
FJ160340; Surface 1; uncultured alpha proteobacterium; Brackish
HQ687798; Surface 1; uncultured bacterium; Marine
FJ160304; uncultured marine alpha proteobacterium; Brackish
FJ825786; Surface 1; Candidatus Pelagibacter; Marine shallow
SAR11clade_SAG1_00150 SAG
CP000699; Sphingomonas wittichii RW1
0.2

**Figure S4e.** SAR11 clade SSU tree (1000 bp size limit except SAGs, only sequences with annotated origin; usearch 95%;, linsi (auto); BMGE (DNAPAM1:2); IQ-tree (Model TIM+G4; fast bootstrapping, 1000 replicates; Only branches with >50% bootstrap support represented)).

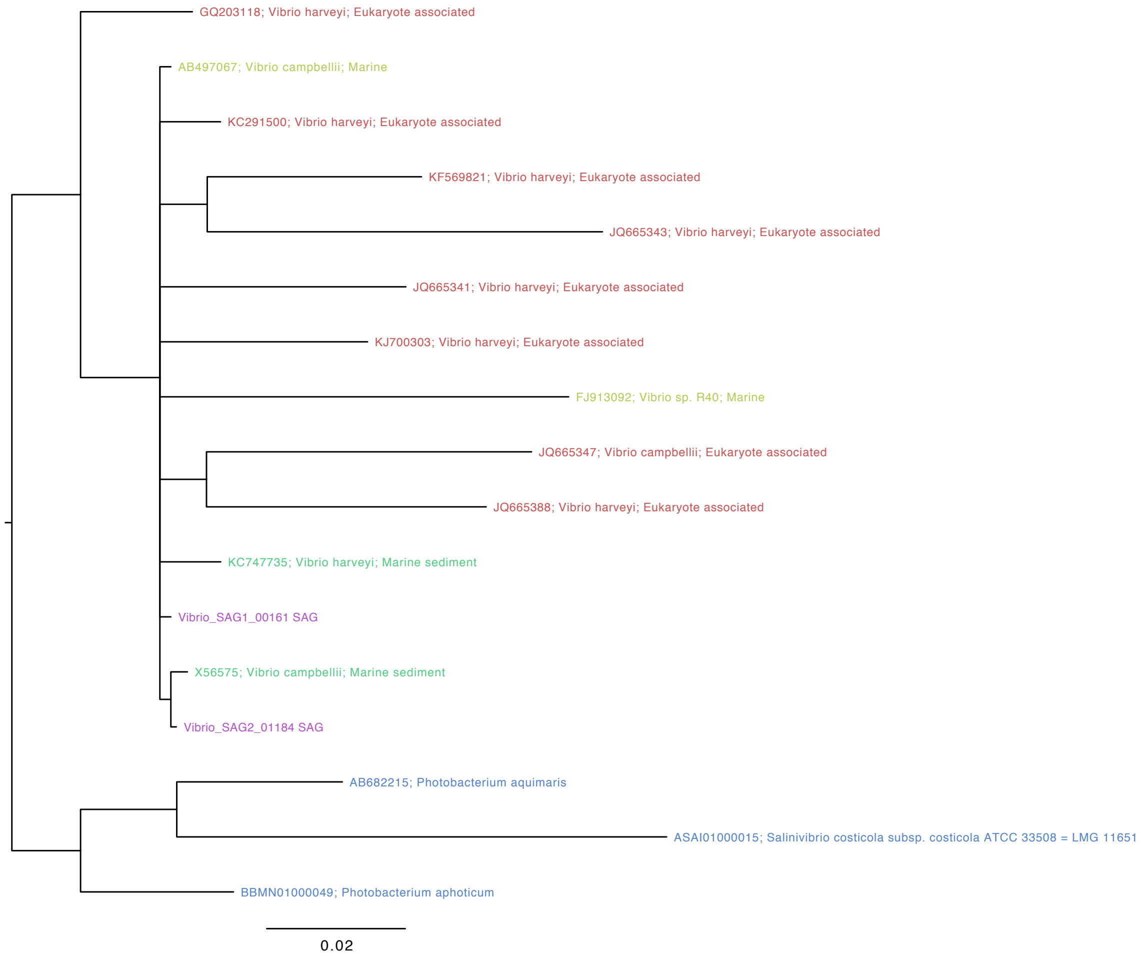


**Figure S4f.** Vibrio harvey clade SSU tree (1000 bp size limit except SAGs, only sequences with annotated origin; usearch 95%;, linsi (auto); BMGE (DNAPAM1:2); IQ-tree (Model HKY+G4; fast bootstrapping, 1000 replicates; Only branches with >50% bootstrap support represented)).

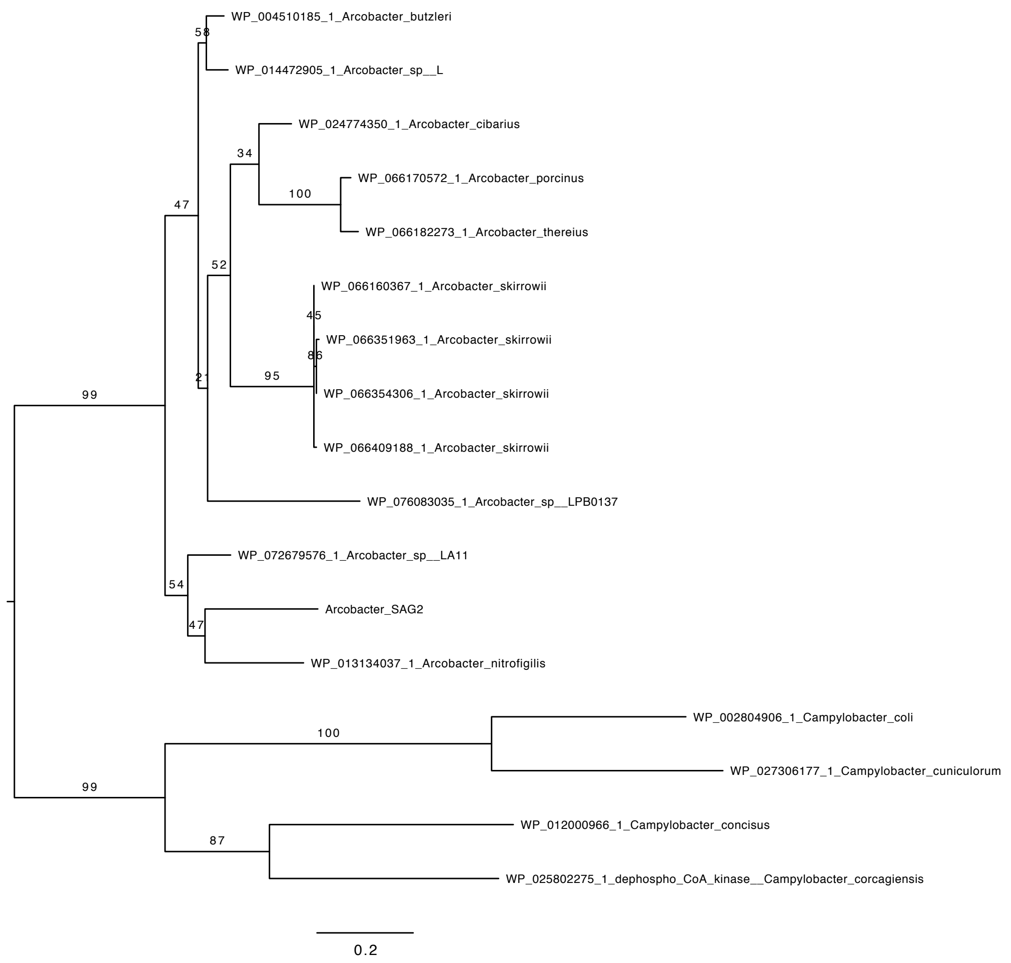


**Figure S4g.** Arcobacter CoA tree (linsi (auto); BMGE (BLOSUM30); IQ-tree (Model cpREV+F+G4; fast bootstrapping, 1000 replicates)).

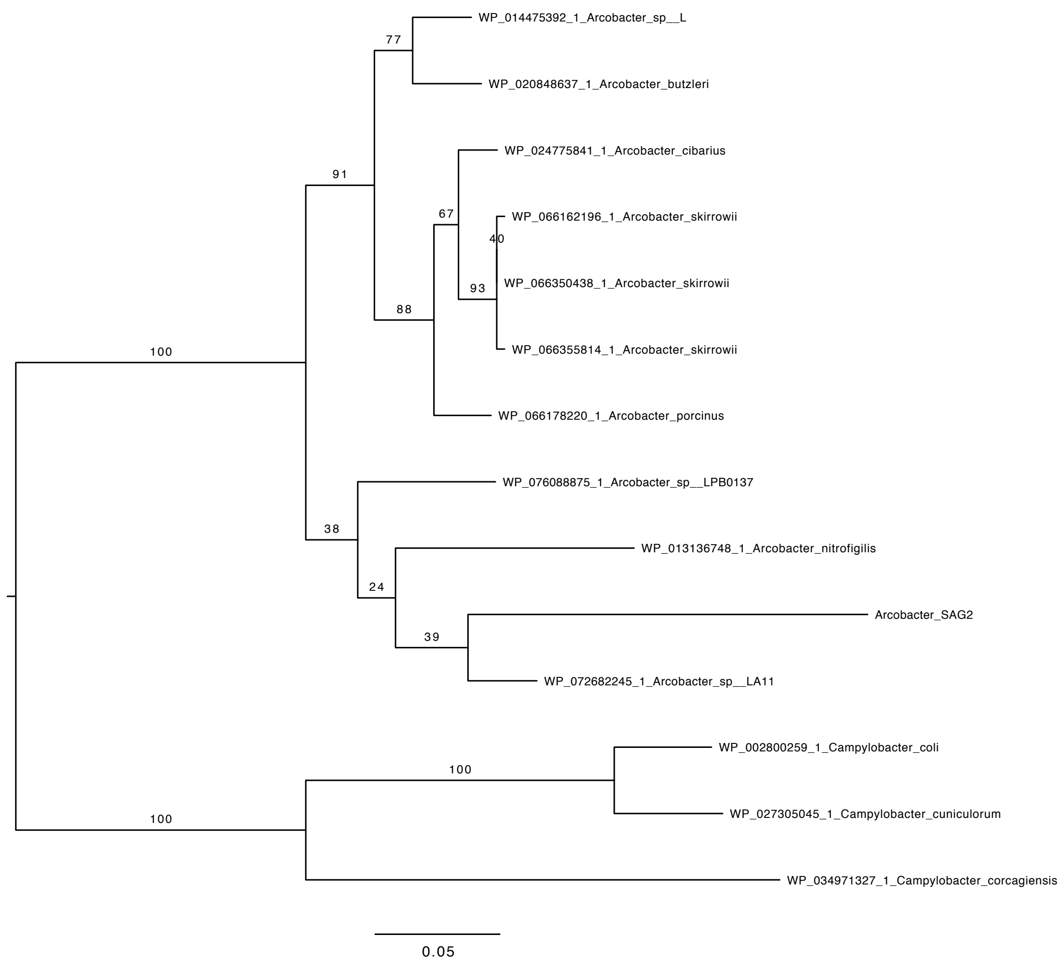


**Figure S4h.** Arcobacter RecA tree (linsi (auto); BMGE (BLOSUM30); IQ-tree (Model LG+G4; fast bootstrapping, 1000 replicates)).

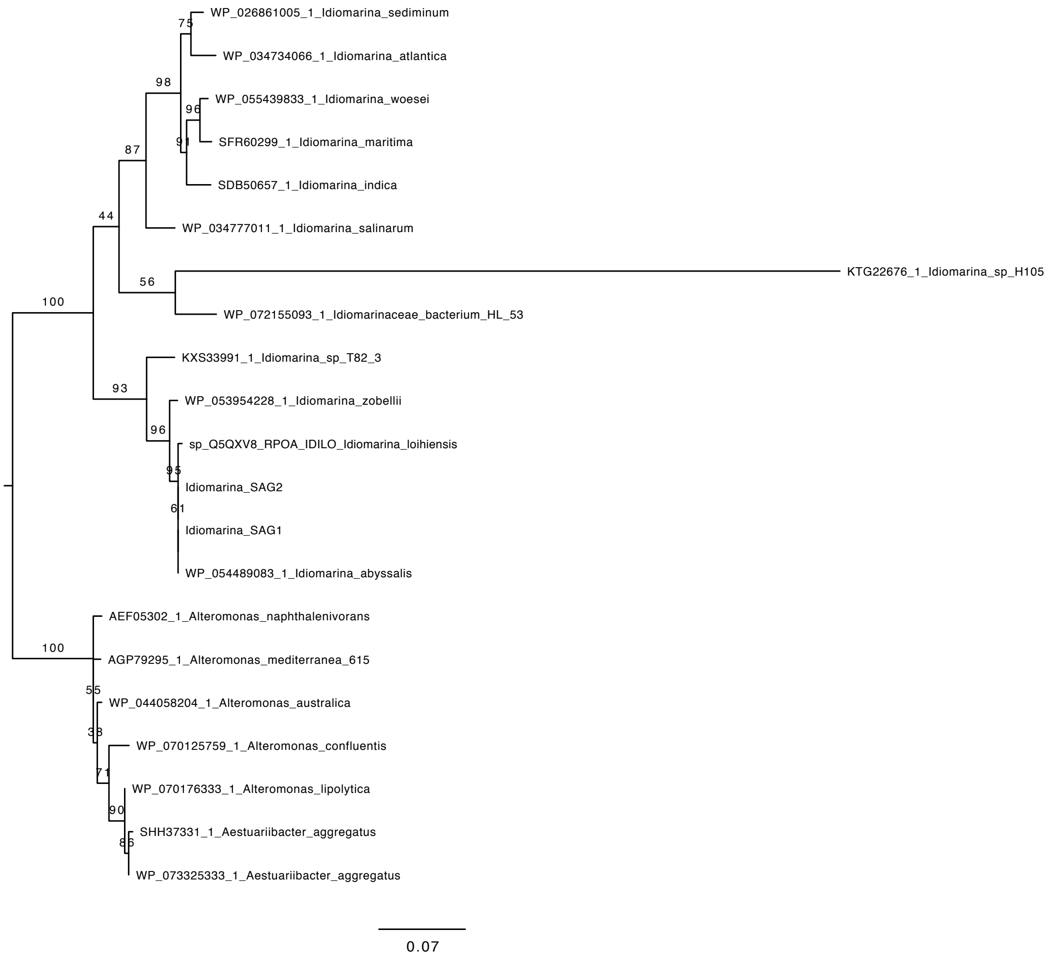


**Figure S4i.** Idiomarina RpoA tree (linsi (auto); BMGE (BLOSUM30); IQ-tree (Model LG+G4; fast bootstrapping, 1000 replicates)).

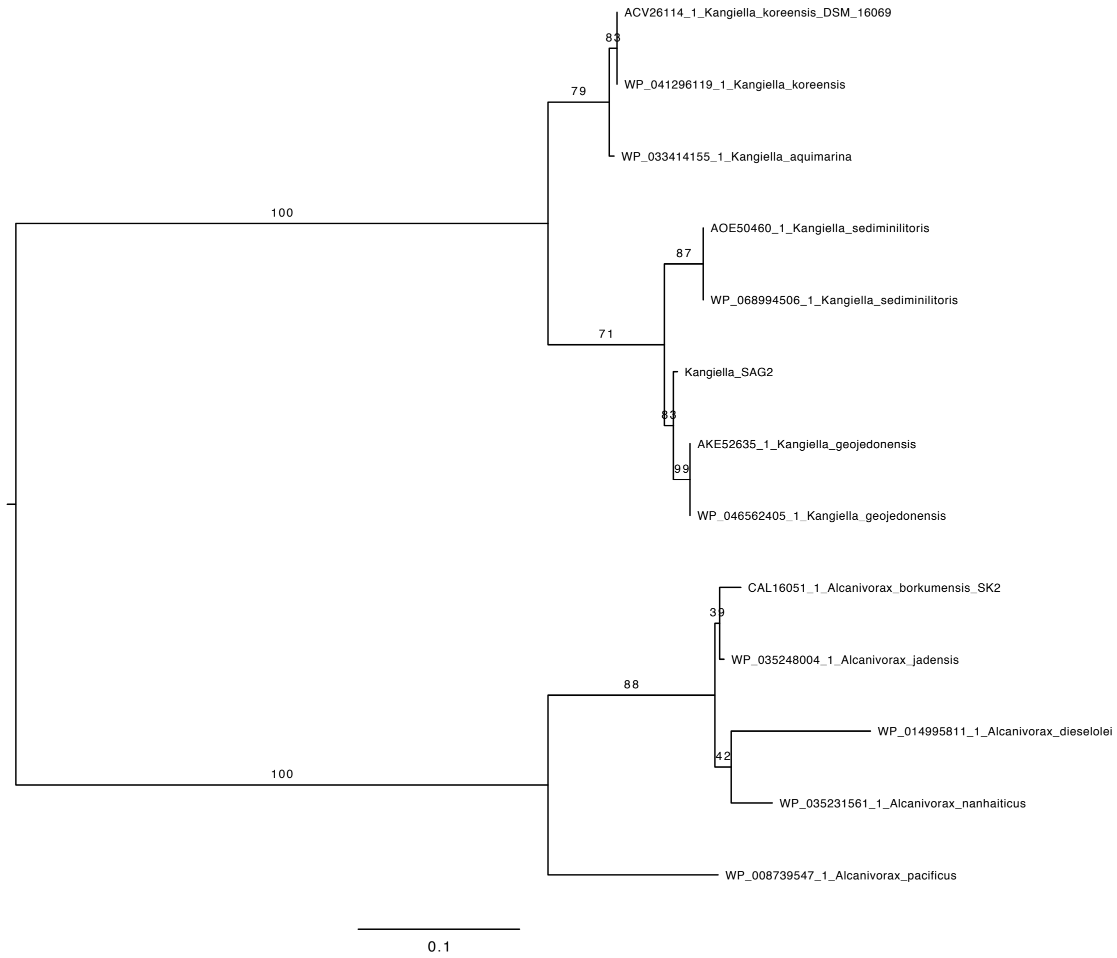


**Figure S4j.** Kangiella FtsZ tree (linsi (auto); BMGE (BLOSUM30); IQ-tree (Model LG+G4; fast bootstrapping, 1000 replicates)).

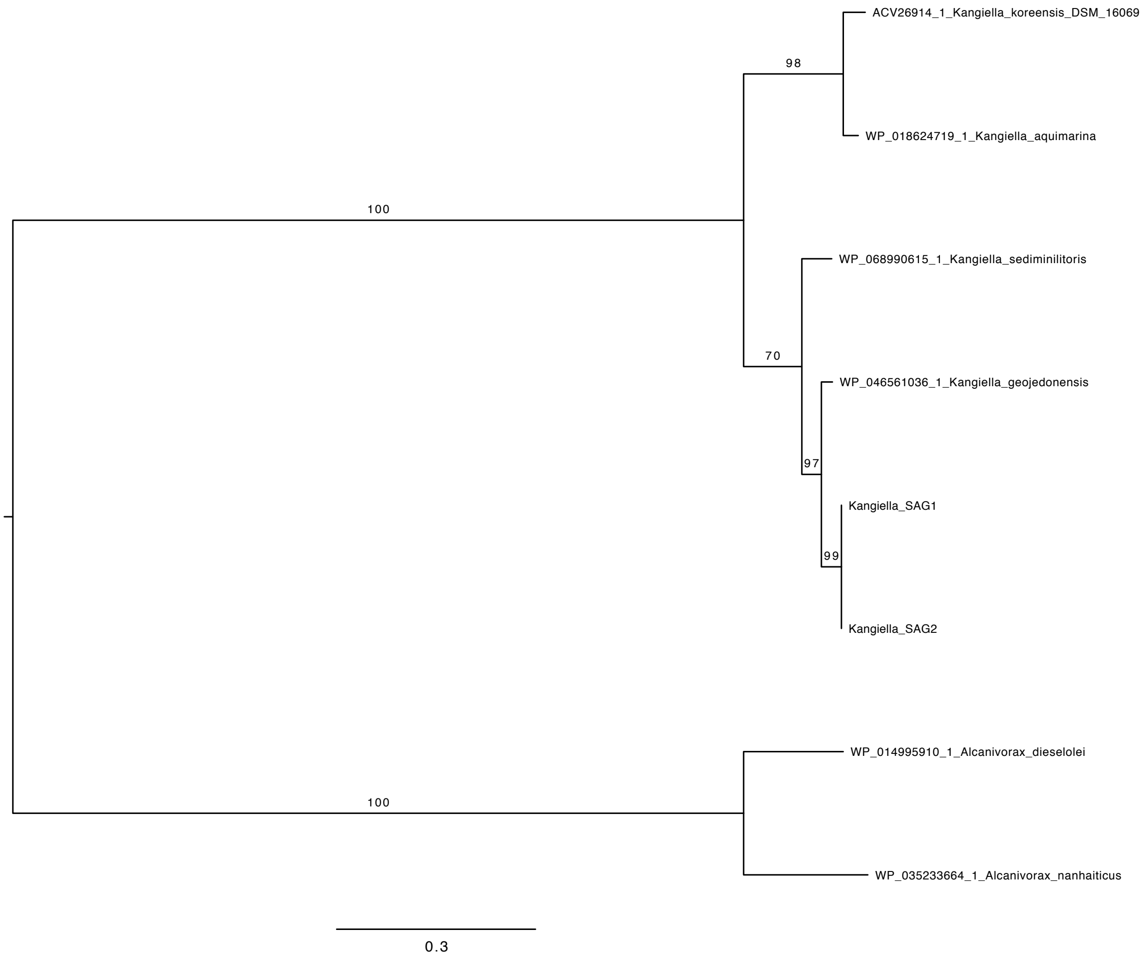


**Figure S4k.** Kangiella HscA tree (linsi (auto); BMGE (BLOSUM30); IQ-tree (Model LG+G4; fast bootstrapping, 1000 replicates)).

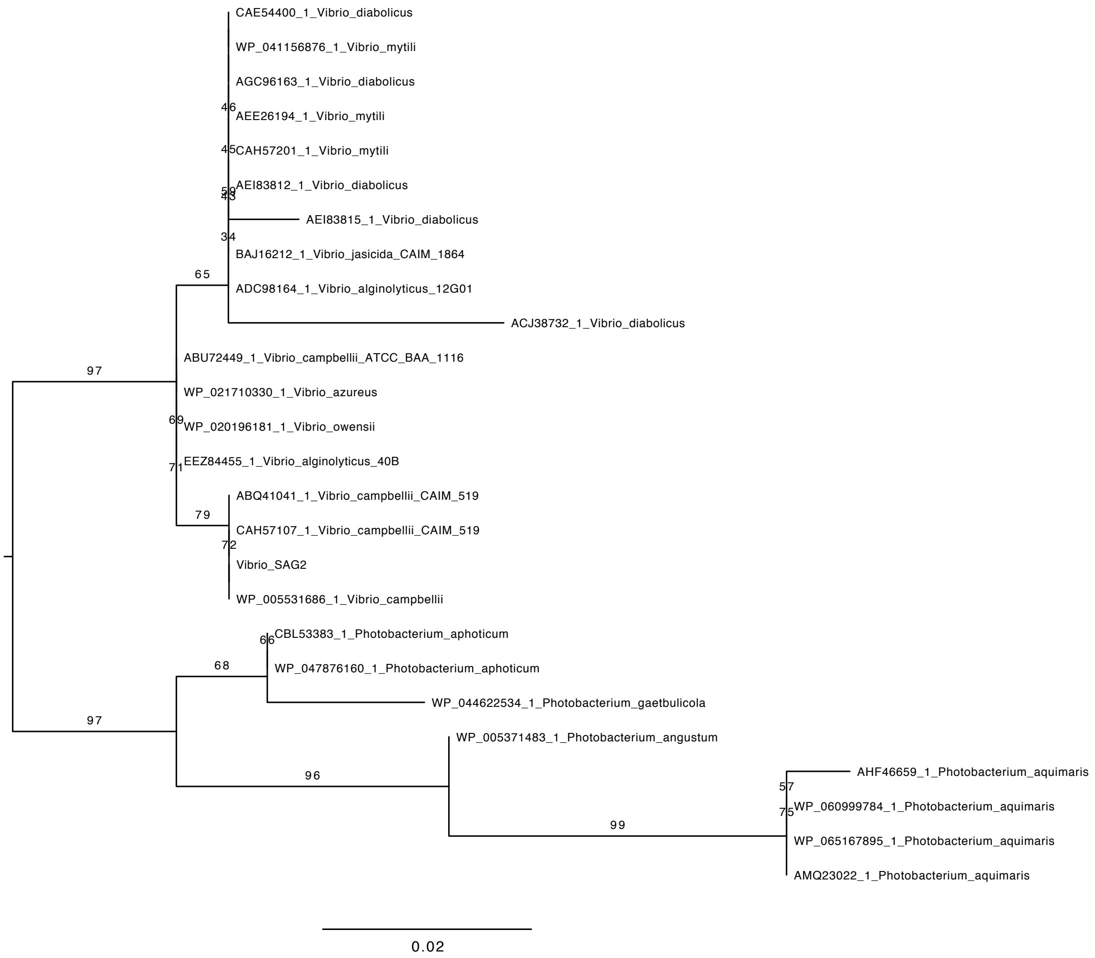


**Figure S4l.** Vibrio RecA tree (linsi (auto); BMGE (BLOSUM30); IQ-tree (Model LG+I; fast bootstrapping, 1000 replicates)).

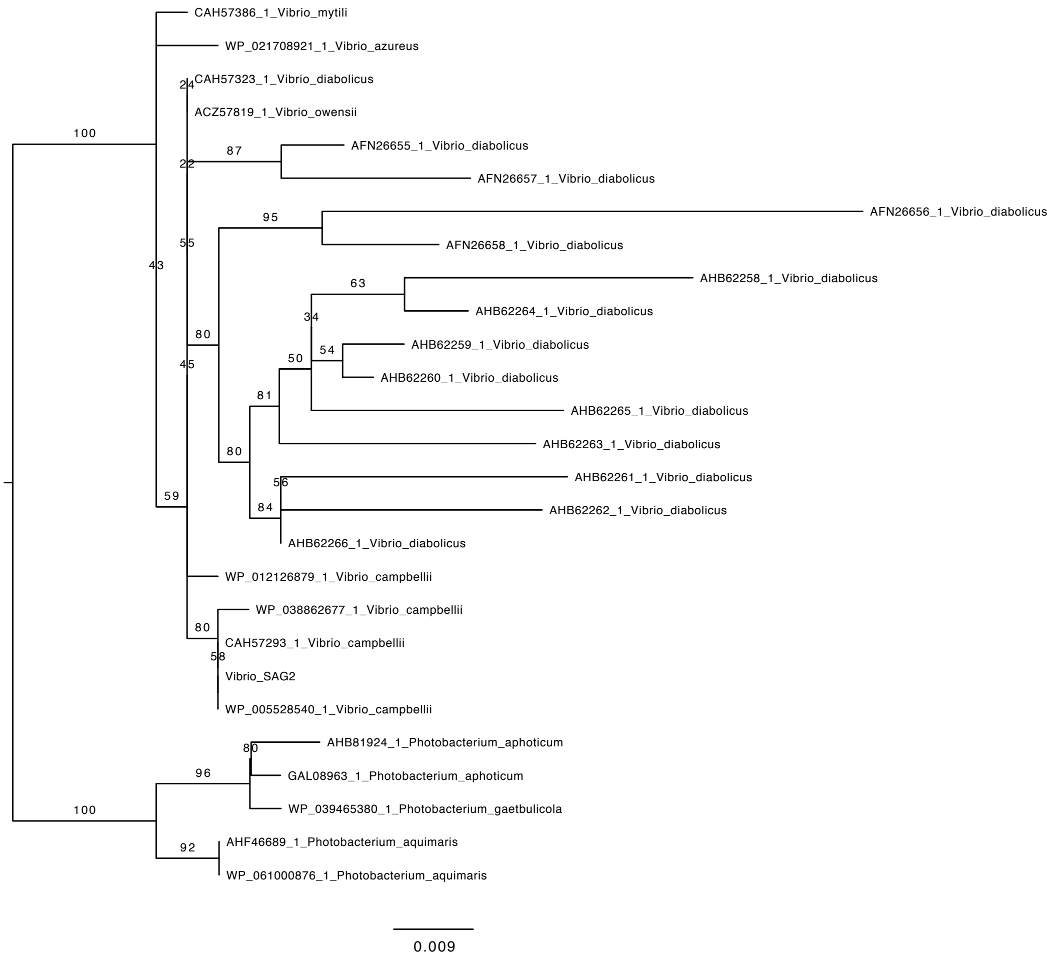


**Figure S4m.** Vibrio RpoA tree (linsi (auto); BMGE (BLOSUM30); IQ-tree (Model JTT+G4; fast bootstrapping, 1000 replicates)).

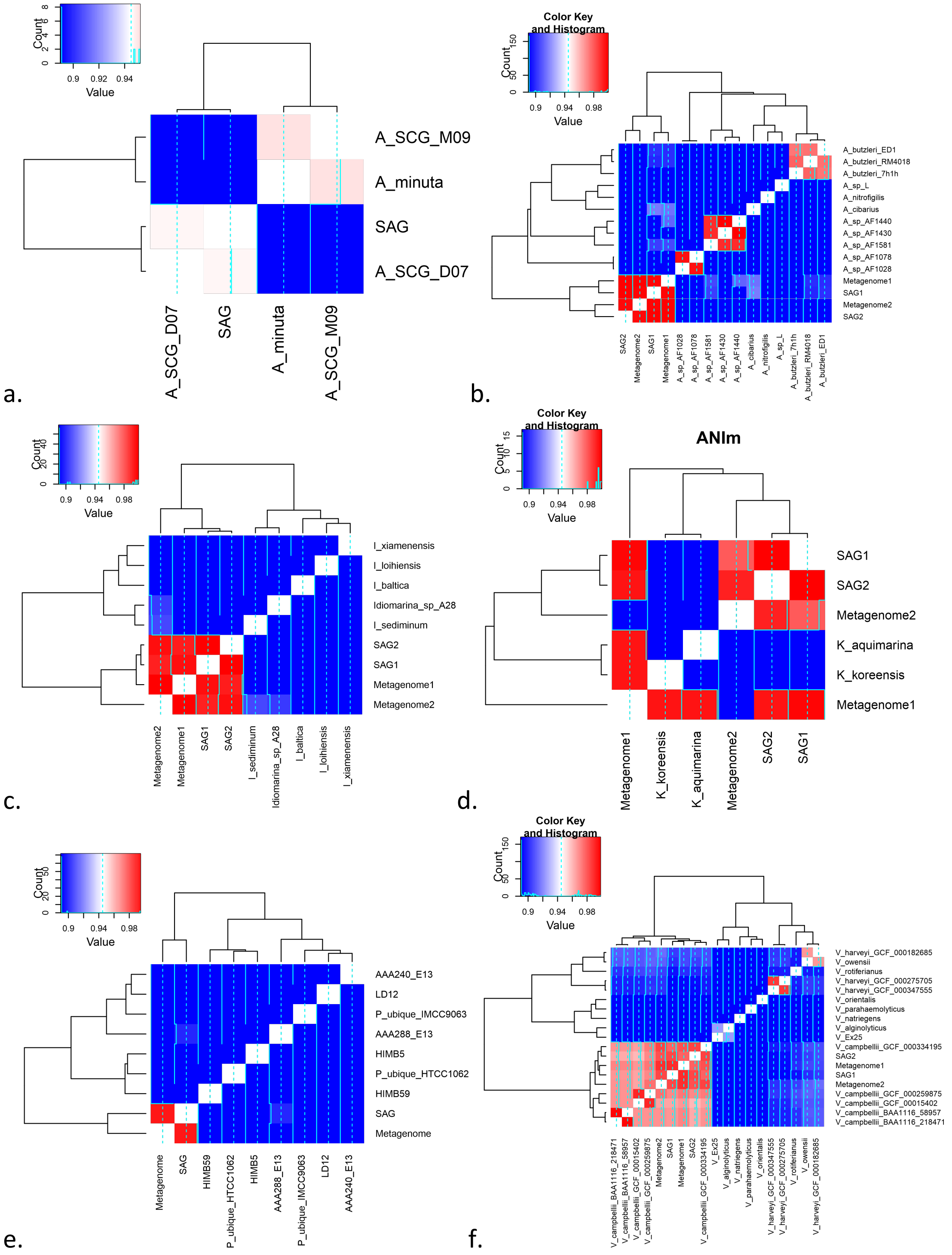


**Figure S5.** Average Nucleotide (ANI) clusters to related reference genomes. Vibrio SAGs have a high level of similarity to *Vibrio campbellii*. Comparison made with the script pyani (https://github.com/widdowquinn/pyani).

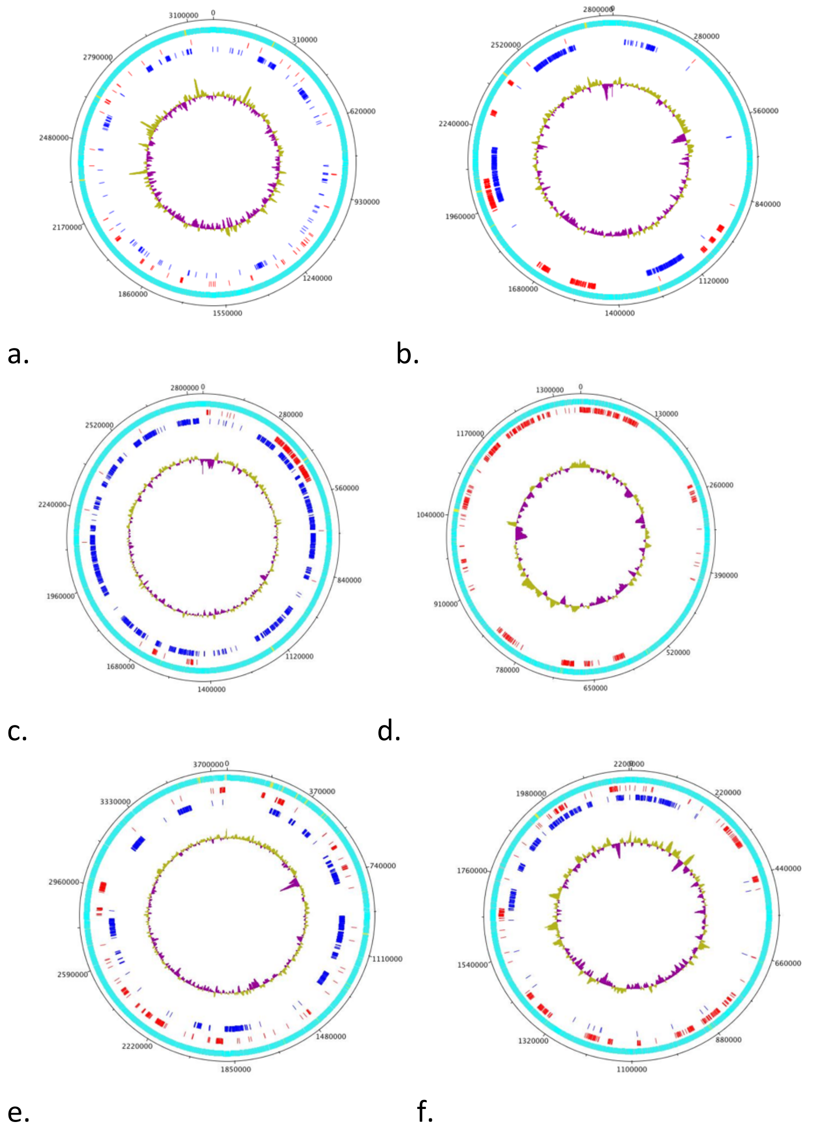


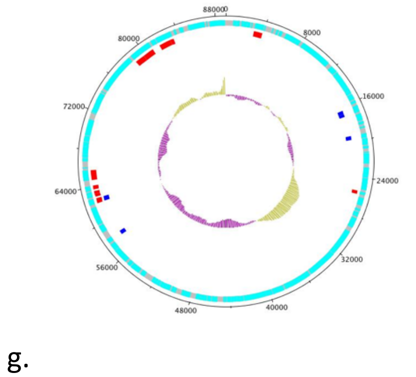


**Figure S6.** Homologous genes (Best reciprocal blast hits) between the SAGs and closely related reference genomes. a.) *Arcobacter nitrofigilis* (NC_014166) b.) *Idiomarina loihiensis* (NC_021286) c.) *Kangiella koreensis* (NC_013166) d.) alpha proteobacteria HIMB5 (NC_018643) e.) *Vibrio campbellii* BAA1116 Chromosome 1(NC_009783) f.) Chromosome 2 (NC_009784) and g.) plasmid (NC_009777). Reference genes are Cyan, SAG1 genes are Red, SAG2 genes are Blue. The center circle indicates Reference GC content with high GC in Green and low GC in purple. Figures made with Artemis (http://www.sanger.ac.uk/science/tools/artemis)

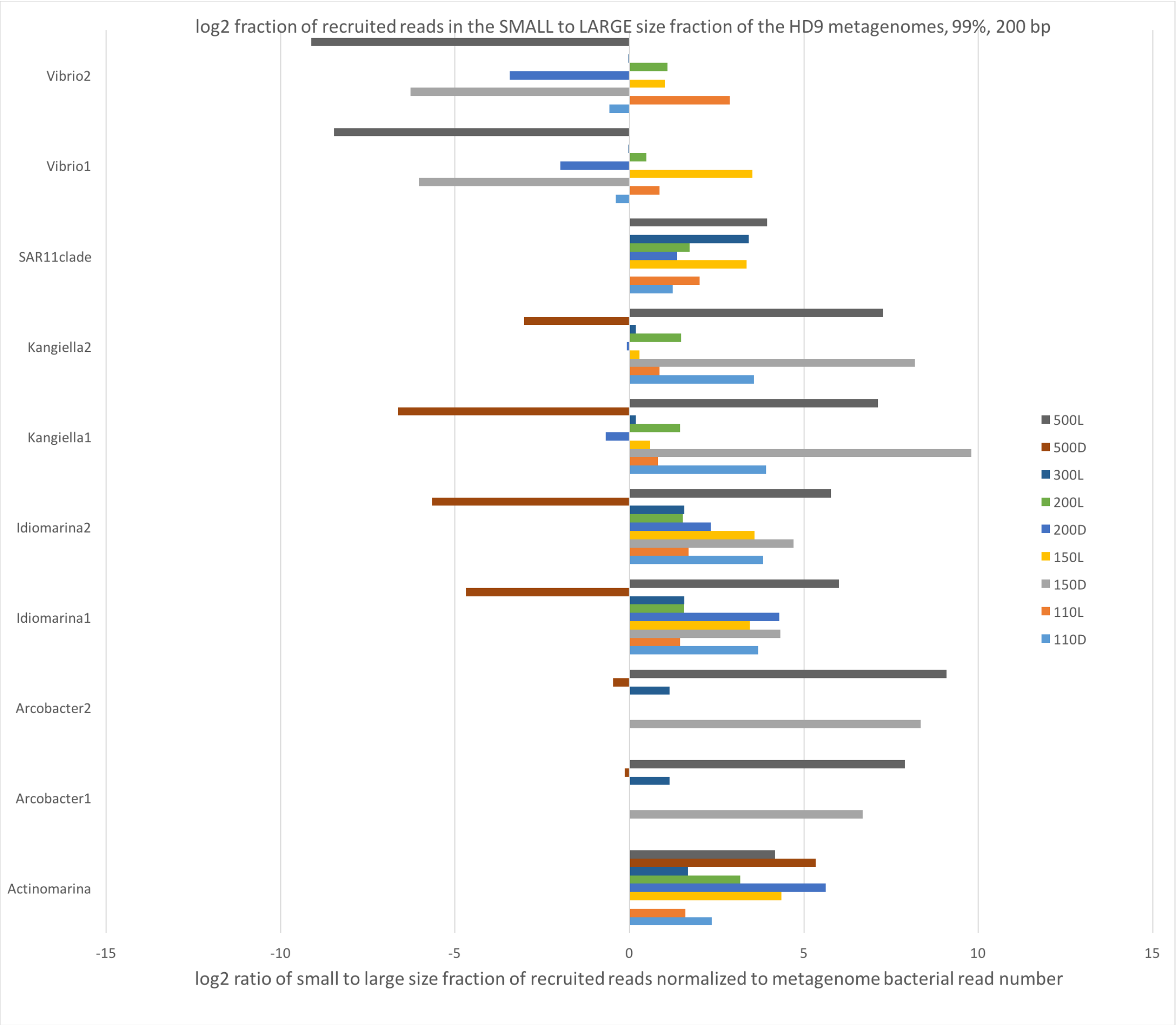


**Figure S7a.** Log2 difference of the ratio between small and large fraction of proportion of bacterial reads recruited by SAGs with high stringency (cutoff e-10, >200 bp, 99% blast hit similarity) in the same trap metagenome (with the ribosomal RNA reads excluded) as the SAGs were isolated. Both Arcobacter and Vibrio SAGs recruited a higher proportion of bacterial reads in the large fraction metagenome than the small, and the other SAGs recruited a higher proportion of bacterial reads in the smaller fraction metagenome than the large. The SAR11 clade SAG did not recruit any reads that met the selection criteria in either of the size fraction metagenomes.

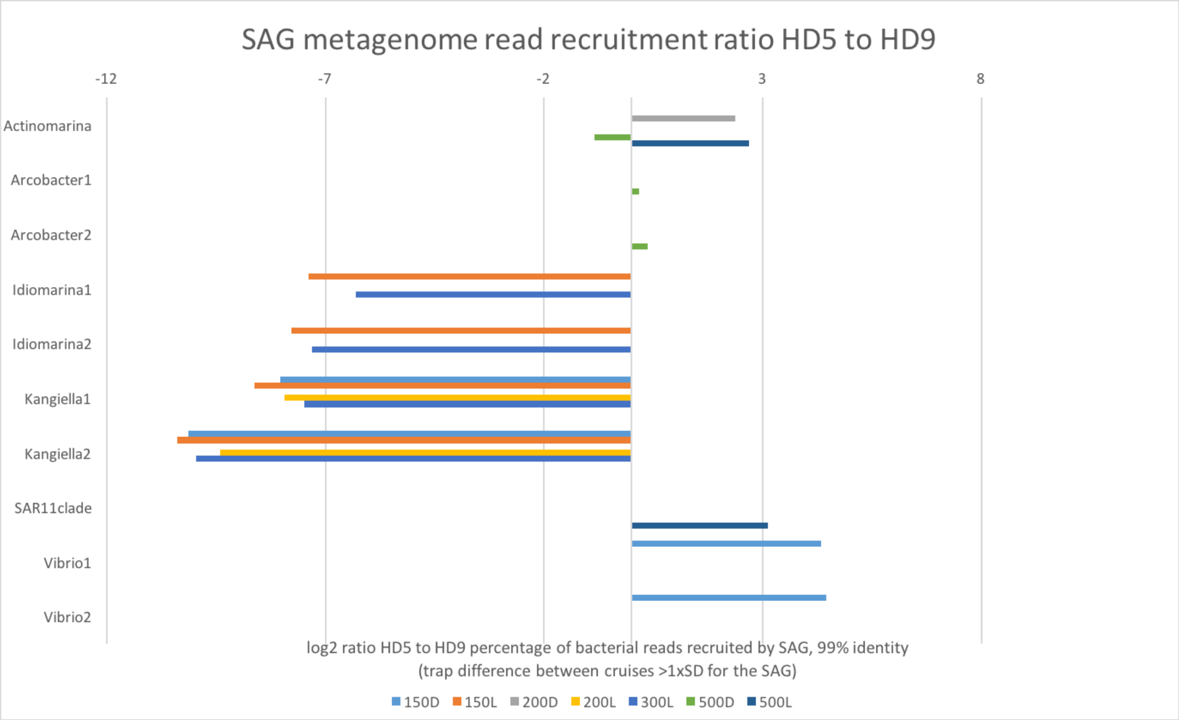


**Figure S7a.** Log2 difference of the ratio between proportion of bacterial reads recruited by SAGs with high stringency (cutoff e-10, >200 bp, 99% blast hit similarity) in pairwise comparable sediment traps (with the ribosomal RNA reads excluded) between the HD5 and the HD9 cruise. Only traps where the difference between cruises exceeded the standard deviation for that SAG is included. Both Arcobacter and Vibrio SAGs recruited a higher proportion of bacterial reads in the HD5 metagenomes than the HD9, and Idiomarina and Kangiella recruited a higher proportion of bacterial reads from the HD9 than the HD5 cruise. Actinomarina and SAR11 more often recruited a higher number of reads from the HD5 cruise, even if there was an exception for Actinomarina for the HD9 trap where it was recruited.

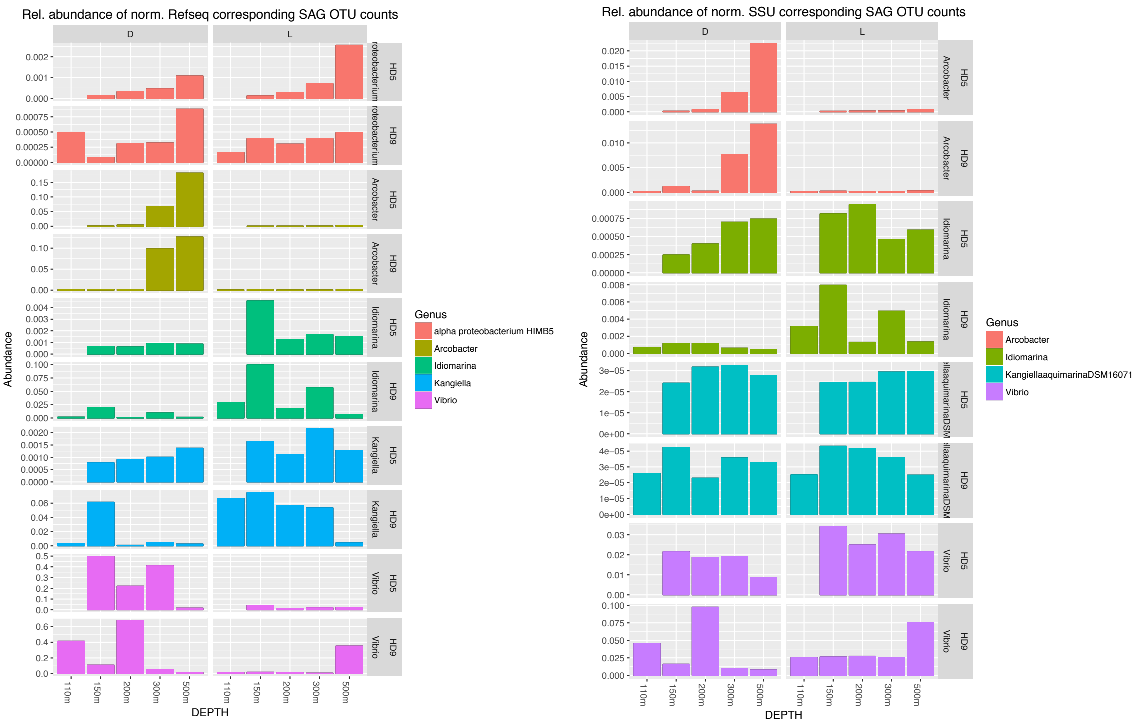


**Figure S8a.** Metagenomeic distribution of OTUs corresponding to SAGs, REFSEQ AND SSU

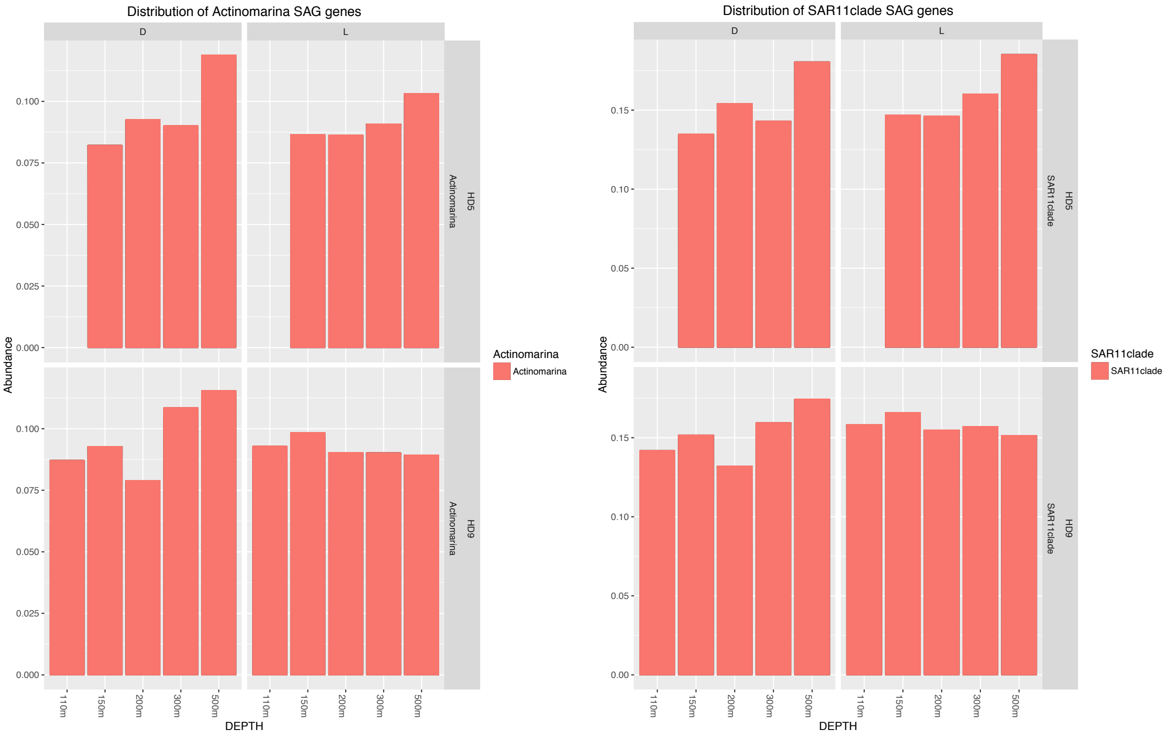


**Figure S8b.**Metagenome distribution of KO's from the Actinomarina and SAR11clade SAGs

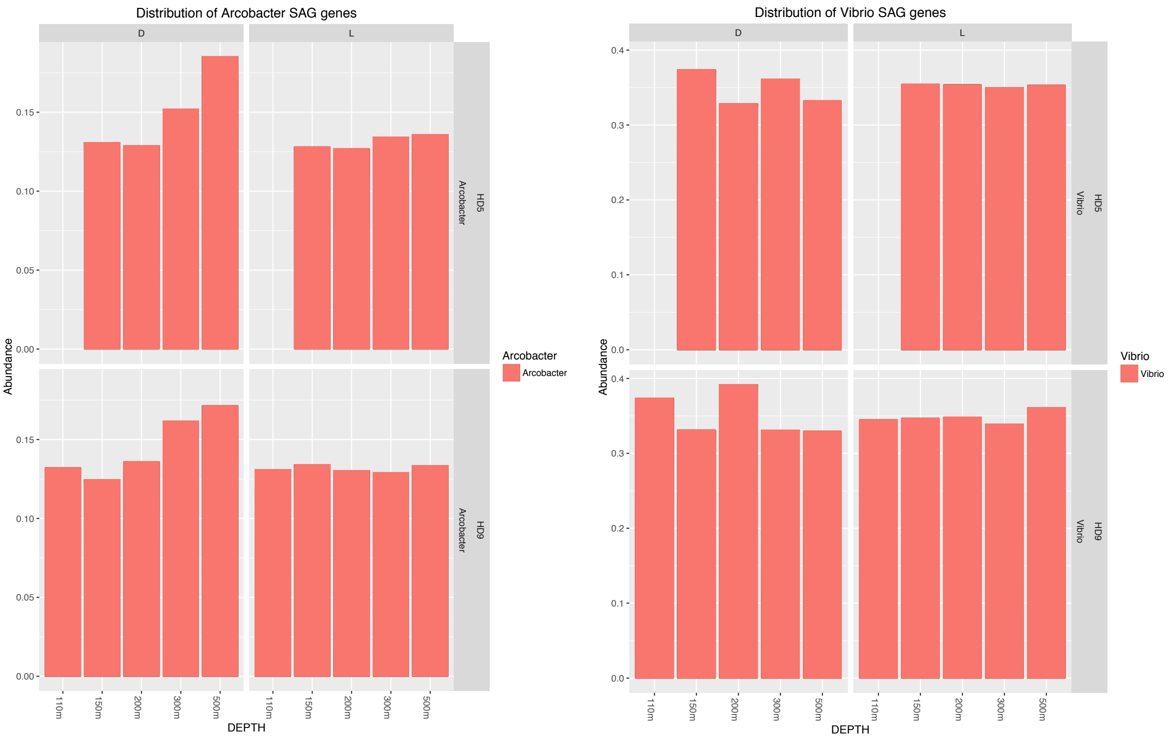


**Figure S8c.** Metagenome distribution of KO's from the Arcobacter and Vibrio SAGs

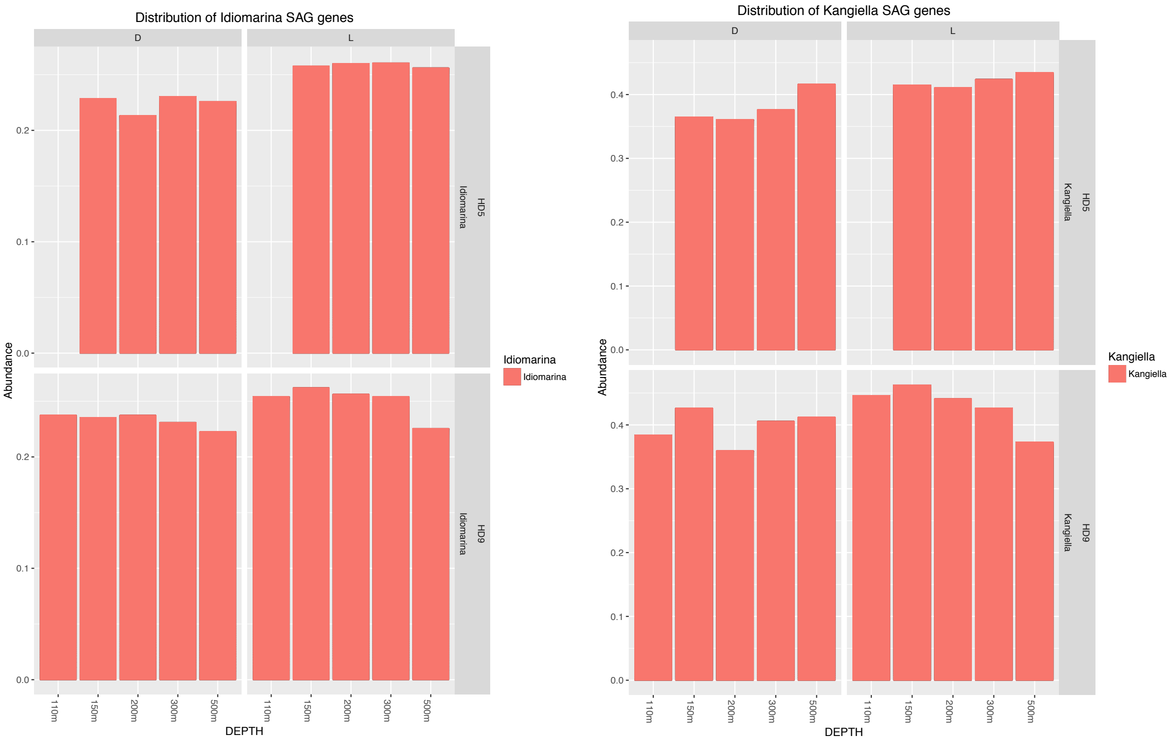


**Figure S8d.**Metagenome distribution of KO's from the Idiomarina and Kangiella SAGs

**a.**

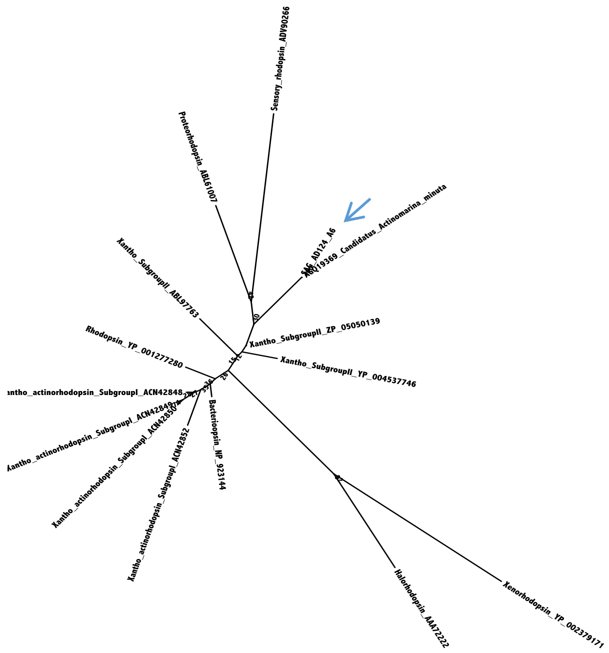


**b.**

```
PROKKA_00295                              ------------------------MEELTYRLFMV----ATVGMLAGTVFLLASSREVK-
gi|523264902|gb|AGQ19369.1|               ------------------------MEELTYRLFMV----ATVGMLAGTVFLLASSREVK-
gi|10580964|gb|AAG19772.1|                --------MLELLPTAVE---GVSQAQITGRPEWIWLALGTALMGLGTLYFLVKGMGVSD
gi|9971913|gb|AAG10475.1|AF279106_37      MKLLLILGSVIALPTFAAGGGDLDASDYTGVSFWL----VTAALLASTVFFFVERDRVS-
                                                                    : *     :     *. :  .*::::..   *.

PROKKA_00295                              PEHRRGVYISALVCGIAWYHYQKMGASWE--------SGAYDTGLRYVDWVLTVPLMFVE
gi|523264902|gb|AGQ19369.1|               PEHRRGVYISALVCGIAWYHYQKMGASWE--------SGSYDTGLRYVDWVLTVPLMFVE
gi|10580964|gb|AAG19772.1|                PDAKKFYAITTLVPAIAFTMYLSMLLGYGLTMVPFGGEQNPIYWARYADWLFTTPLLLLD
gi|9971913|gb|AAG10475.1|AF279106_37      AKWKTSLTVSGLVTGIAFWHYMYMRGVWIE-------TGDSPTVFRYIDWLLTVPLLICE
                                           . :    :: ** .**:  *  *   :                ** **::*.**:: :

PROKKA_00295                              VLAVTRKGAAY-NEAVRNWGVAATVMIGAGYYGETSVAGS-NEYWTGFVIAMATYIWLMR
gi|523264902|gb|AGQ19369.1|               VLAVTRKGAAY-NEAVRNWGIAATVMIGAGYYGETSAAGS-NEYWTGFVIAMATYVWLMR
gi|10580964|gb|AAG19772.1|                LALLVDADQGT----ILALVGADGIMIGTGLVGALTKVYSYRFVW--WAISTAAMLYILY
gi|9971913|gb|AAG10475.1|AF279106_37      FYLILAAATNVAGSLFKKLLVGSLVMLVFGYMGEA---GI-MAAWPAFIIGCLAWVYMIY
                                          .  :           .     .  :*:  *  *           *  : *.  : ::::

PROKKA_00295                              NLQAE-GEGL----KGDEAAAFENIKNLILVGWIIYPLGYIAPVA---GDFDAIREVLYT
gi|523264902|gb|AGQ19369.1|               NLQAE-GEGL----KGDQAVAFENIKNLILVGWIIYPLGYIAPVV---GDFDAIREVLYT
gi|10580964|gb|AAG19772.1|                VLFFGF-TSKAESMRPEVASTFKVLRNVTVVLWSAYPVVWLIGSEGAGIVPLNIETLLFM
gi|9971913|gb|AAG10475.1|AF279106_37      ELWAGEGKSACNTASPAVQSAYNTMMYIIIFGWAIYPVGYFTGYLMGDGGSALNLNLIYN
                                           *      .           ::: :  : :. *  **: ::                :::

PROKKA_00295                              IADIINKVGLGVLVLQMARVKSGEKVS*------------
gi|523264902|gb|AGQ19369.1|               IADIINKVGLGVLVLQMARVQSGEKVS-------------
gi|10580964|gb|AAG19772.1|                VLDVSAKVGFGLILLRSRAIFGEAEAPEPSAGDGAAATSD
gi|9971913|gb|AAG10475.1|AF279106_37      LADFVNKILFGLIIWNVAVKESSNA---------------
                                          : *.  *: :*::: .     .
```

**Figure S9a.** The rhodopsin from the Actinomarina SAG clusters closely with the Actinomarina rhodopsin as the MAC-rhodopsin cluster (AGQ19369, Ghai et al., 2013). B) They share amino acids at position 85, 89, 93 and 96. The D, T and E at position 85, 89 and 96 indicate function as a proton pump (Yoshizawa *et al.,* 2014) and the M at position 93 suggest adaptation to green light (Furhman *et al.,* 2008).

Numbering from *Halobacterium salinarum* bacteriorhodopsin AAG19772.1 (Yoshizawa *et al.,* 2014). Pos. 93 in corresponds to 105 in proteorhodopsin from clone eBAC31A08 (AAG10475, Man *et al.,* 2003).

**Supplemental references**


Fuhrman, J. a, Schwalbach, M. S., and Stingl, U. (2008). Proteorhodopsins: an array of physiological roles? *Nat. Rev. Microbiol.* 6, 488–94. doi:10.1038/nrmicro1893.

Man, D., Wang, W., Sabehi, G., Aravind, L., Post, A. F., Massana, R., et al. (2003). Diversification and spectral tuning in marine proteorhodopsins. *EMBO J.* 22, 1725–1731. doi:10.1093/emboj/cdg183.

Yoshizawa, S., Kumagai, Y., Kim, H., Ogura, Y., Hayashi, T., Iwasaki, W., et al. (2014). Functional characterization of flavobacteria rhodopsins reveals a unique class of light-driven chloride pump in bacteria. *Proc. Natl. Acad. Sci.* 111, 6732–6737. doi:10.1073/pnas.1403051111.
